# Gender‐Specific Depression‐Anxiety Symptom Networks and the Impact of Weight Status: Insights From a Large‐Scale Study

**DOI:** 10.1002/pchj.70104

**Published:** 2026-05-29

**Authors:** Meng‐Bi Yang, Yi‐Xuan Wu, Wei‐Xia Zhang, Hui‐Ying Liu, Han Lin, Min Xi, Shu‐Bin Si

**Affiliations:** ^1^ School of Mechanical Engineering, Northwestern Polytechnical University Xi'an China; ^2^ Department of Psychology University of California, Los Angeles Los Angeles California USA; ^3^ Department of Physical Education Northwestern Polytechnical University Xi'an China; ^4^ School of Engineering Audit, Nanjing Audit University Nanjing China; ^5^ Key Laboratory of Biomedical Information Engineering of Ministry of Education Xi'an China; ^6^ Hospital of Northwestern Polytechnical University, Northwestern Polytechnical University Xi'an China; ^7^ Key Laboratory of Internet of Things‐Enabled Manufacturing and Quality Control Technology for Aeronautical Equipment, Ministry of Industry and Information Technology Xi'an China

**Keywords:** anxiety, BMI, Chinese adults, depression, gender difference, network analysis

## Abstract

The relationship between body mass index (BMI), gender, and specific depression‐anxiety symptoms remains unclear. This study examined how BMI and gender are associated with depression‐anxiety symptom networks in 9091 Chinese adults aged 19–65 years using the Patient Health Questionnaire‐9 (PHQ‐9) and the Generalized Anxiety Disorder‐7 (GAD‐7) scales. Participants were categorized into four BMI subgroups: underweight, normal weight, overweight, and obesity groups. Network analysis identified core symptoms, including “motor problems” (PHQ8), “uncontrollable worry” (GAD2), and “trouble relaxing” (GAD4). Differences in network structure were observed across BMI groups, with males showing variations between the underweight, normal weight, and obesity groups, and females demonstrating significant differences in both network structure and global strength between the underweight and overweight groups. Gender differences were also found in the global strength of the overall network and in the network structure of the normal weight group. Limitations include potential residual confounders, the cross‐sectional design, reliance on self‐reported data, and unbalanced sample sizes. These findings suggest that BMI may be associated with the depression‐anxiety symptom network in Chinese adults, with distinct gender‐specific patterns, highlighting the importance of considering both BMI and gender in developing targeted mental health interventions.

## 1 Introduction

Weight status, closely related to body appearance and shape, has become a global concern (Puhl and Heuer 2010). Deviations from normal weight status may pose both physiological and mental health risks (Li et al. 2023). In the evaluation of weight status, body mass index (BMI) is accepted as an internationally recognized standard (Khazanov and Ruscio 2016). Using BMI as an indicator of weight status, empirical studies have unveiled a complex U‐shaped association between weight status and mental health, suggesting high risks of depression and anxiety for underweight and overweight individuals (He et al. 2022; Ma et al. 2021; Yeom and Kim 2022). Individuals who are chronically underweight are at an increased risk of developing osteoporosis (Park et al. 2023), impaired immune function (Dobner and Kaser 2018) and an elevated likelihood of premature mortality (Roh et al. 2014). Males with low BMI exhibited a 12% elevated risk of suicide compared to those within the normal BMI range. This phenomenon was attributed to the observed reduction in the levels of neurotransmitters in the brain associated with feelings of euphoria (Smith et al. 2009). Meanwhile, those who are overweight or obese may be more susceptible to depression and anxiety due to stigmatization, discrimination, and rejection (Kivimäki et al. 2009; Puhl and Heuer 2010).

The relationship between weight status and mental health demonstrates considerable disparities across gender and culture (van den Broek et al. 2018; Warnick et al. 2022; Xie et al. 2023). In UK adults, the risk of mental disorders progressively increased with increasing BMI in females, whereas males displayed a U‐shaped association, in which both underweight and overweight individuals were at higher risk (McCrea et al. 2012). This gender disparity has been further corroborated by research conducted in Japan (Hori et al. 2016). However, such a pattern was not replicated in China, as overweight males exhibited an elevated risk of depression, whereas this association was not statistically significant in females (Xie et al. 2023). These discrepancies underscore the intricate nature of the relationship between BMI and mental health, and the association may be shaped by a complex interplay of cultural and social factors (Xie et al. 2023; Sunwoo et al. 2011).

Within the unique socio‐cultural context of contemporary China, weight status is not merely a physiological indicator but a socially embedded construct, shaped by gendered body ideals and role expectations. Thinness is often emphasized for women and associated with attractiveness, self‐discipline, and social approval, whereas men are more frequently evaluated based on strength, competence, and their capacity to fulfill familial and social responsibilities (Tang et al. 2024). Deviation from these gender‐specific norms may expose individuals to different forms of social scrutiny, stigma, and internalized dissatisfaction. Furthermore, traditional norms surrounding emotional restraint, particularly among men, may influence how psychological distress is experienced and expressed (Gualdi‐Russo et al. 2022). Under such cultural conditions, BMI‐related pressures may not only affect the severity of depression and anxiety, but also shape the pattern through which specific symptoms co‐occur and interact. Consequently, examining BMI and gender jointly is theoretically critical in understanding the culturally embedded structure of psychological distress in Chinese adults.

Previous studies have primarily focused on the relationship between BMI and total scores for depression or anxiety. However, depression and anxiety are heterogeneous disorders with diverse symptoms, and reliance on total scores may obscure important symptom‐level information (Bai et al. 2022; Fried et al. 2014, 2017; Fried and Nesse 2015; Li et al. 2023, 2024; Ramos‐Vera et al. 2022). Thus, there remains a lack of insight into the relationship between BMI and symptoms of depression and anxiety (Li et al. 2023; Ramos‐Vera et al. 2022). Moreover, most studies have focused on narrow BMI ranges, such as obesity and overweight (Kim et al. 2015; Noh et al. 2015), or confined their analyses to specific populations, such as males (Liao et al. 2020), females (Chen et al. 2020), college students (Li et al. 2024), adolescents (Chen, Zhang, et al. 2024), and the elderly (Lotfaliany et al. 2019). Research examining the full BMI spectrum in broader adult populations remains limited. Therefore, beyond overall symptom severity, it remains unclear how BMI and gender are associated with specific depression‐anxiety symptoms.

Network analysis is an effective tool for investigating the complex relationships among specific symptoms of depression and anxiety across different BMI and gender groups (Borsboom 2017; Fried et al. 2014, 2017; Hou et al. 2023; Si et al. 2023). This method has been widely used in psychiatry research (Fried et al. 2020; Liang et al. 2023; Ramos‐Vera et al. 2022; Wang, Wu, et al. 2024). By constructing behavior‐symptom networks and calculating node centrality, prior work has identified core symptoms linking obesity‐related eating behaviors with depression‐anxiety symptoms (Wang, Wu, et al. 2024). Previous studies have shown that core symptoms are vital targets for intervention (Kaiser et al. 2021; Yang et al. 2024; Zhang et al. 2024). From a BMI‐gender interaction perspective, this approach is particularly valuable, as culturally embedded weight‐related stressors may activate different symptom pathways across genders (Xie et al. 2023). For example, females may respond better to interventions targeting emotional eating, while males may benefit more from interventions focused on physical activity (Du et al. 2022; Wang et al. 2022). Thus, identifying core symptoms within each BMI‐gender subgroup may help clarify differential mechanisms of psychological distress and provide evidence for more stratified prevention and intervention strategies.

The present study aimed to examine the interactions between specific symptoms of depression and anxiety in adults across BMI subgroups (underweight, normal weight, overweight, and obesity) and gender subgroups, by using network analysis. We focused on identifying core symptoms and examining group differences in network structure and global strength. By identifying core symptoms and group differences, our findings are expected to provide clinically relevant information for more individualized psychological interventions.

## 2 Methods

### 2.1 Study Population and Sample

This study utilized data from the Psychology and Behavior Investigation of Chinese Residents (PBICR), a nationwide cross‐sectional survey conducted between June 10 and September 15, 2021. The survey spanned 23 provinces, 5 autonomous regions, and 4 centrally administered municipalities in the Chinese mainland. A three‐stage stratified probability sampling methodology was implemented: (a) Primary sampling units: Direct inclusion of provincial capitals, autonomous region capitals, and municipalities (Beijing, Tianjin, Shanghai, Chongqing); (b) secondary sampling: Random selection of 2–6 non‐capital prefecture‐level cities per province/autonomous region, resulting in 120 cities; (c) tertiary sampling: Quota sampling within these cities based on demographic characteristics from the “Seventh National Population Census in 2021” (OotLGotSCftSNP 2021). Quota attributes included gender, age and urban/rural distribution, with the objective of ensuring that the sample matched the national population on these key demographic characteristics. Within each sampled city, participants were recruited according to pre‐specified quota targets, and field investigators monitored recruitment progress to ensure alignment with the census‐based distributions.

Data were collected using a validated structured questionnaire administered through in‐person interviews. For participants with physical limitations hindering independent completion, trained researchers provided one‐on‐one assistance. Logical consistency checks (e.g., implausible anthropometric values or contradictory responses) were applied during data cleaning, and records failing these checks were removed. Detailed sampling procedures have been previously published (Wang et al. 2022).

Individuals were included if he or she (a) was aged 19–65 years. The age range was determined based on the predefined categorical age options available in the database (≤ 18, 19–25, 26–30, 31–35, 36–40, 41–45, 46–50, 51–55, 56–59, 60–65, 66–70, 71–75, 76–80, 81–85, 86–90, 91–95, 96–100, ≥ 101). We excluded individuals aged < 18 years due to developmental differences and school‐related stressors, and those aged ≥ 66 years to reduce the influence of age‐related physical comorbidities and functional decline; (b) was a permanent resident of China (≤ 1 month annual absence); (c) had capacity to provide informed consent and comprehend survey items. Individuals with any of the following criteria were excluded: (a) had diagnosed cognitive impairments or psychiatric disorders; (b) was concurrently participating in similar studies; and (c) declined participation.

The final analytic sample comprised 9091 valid responses. Detailed methodological specifications of PBICR have been previously documented (Hao et al. 2023). Ethical approval was obtained from the Shaanxi Health Culture Research Centre (JKWH‐2021‐01) and Jinan University Ethics Committee (JNUKY‐2021‐018), and permission to use the dataset was obtained by one of the authors.

### 2.2 Measurement

#### 2.2.1 Body Mass Index

Height and weight were obtained through a standardized electronic questionnaire. The questionnaire explicitly instructed participants to report height in centimeters and weight in kilograms. To minimize potential input errors (e.g., misreporting weight units such as “jin” instead of kilograms), the anthropometric data underwent plausibility and logical consistency checks during data cleaning. Records with implausible values were excluded.

Based on prior research (He et al. 2022) and the Chinese adult criteria (WS/T 428‐2013), BMI categories were classified as underweight (BMI < 18.5 kg/m^2^), normal weight (18.5 ≤ BMI < 24.0 kg/m^2^), overweight (24.0 ≤ BMI < 28.0 kg/m^2^), and obesity (BMI ≥ 28.0 kg/m^2^). These cut‐offs are specifically tailored to Chinese populations, as they better reflect obesity‐related risk in Chinese populations than the standard WHO classification (Zhou 2002).

#### 2.2.2 Depression

Depressive symptom severity was measured using the Mandarin version of the Patient Health Questionnaire‐9 (PHQ‐9), a 9‐item instrument aligned with DSM‐IV diagnostic criteria (Chen et al. 2015; Kroenke et al. 2001). Participants rated symptom frequency over the preceding 2 weeks on a 4‐point Likert scale (0 = *not at all* to 3 = *almost daily*), yielding total scores ranging from 0 to 27. Clinical severity was categorized as: minimal (0–4), mild (5–9), moderate (10–14), moderately severe (15–19), and severe (20–27) following established cutoffs. The Chinese adaptation has demonstrated excellent psychometric properties in prior validation studies, and internal consistency in the current sample was excellent (*α* = 0.94).

#### 2.2.3 Anxiety

Anxiety symptoms were evaluated using the Chinese version of the 7‐item Generalized Anxiety Disorder Scale (GAD‐7) (Spitzer et al. 2006; Wang et al. 2018), which is based on the Diagnostic and Statistical Manual of Mental Disorders, Fourth Edition (DSM‐IV). The scale consists of seven items, each assessed on a four‐point Likert scale with the following response options: “not at all” = 0, “a few days” = 1, “more than half of the days” = 2, and “almost every day” = 3. Higher GAD‐7 scores indicate more severe anxiety symptoms, with total scores of 5, 10, 15 and 20 categorized as mild, moderate, moderately severe, and severe anxiety symptoms, respectively. In the present study, Cronbach's alpha for the entire scale of the GAD‐7 was 0.96.

### 2.3 Data Analysis

#### 2.3.1 Traditional Analysis

Firstly, the normality of continuous variables was assessed using the Kolmogorov–Smirnov test. An examination of Q–Q plots indicated that the continuous variables did not follow a normal distribution. Continuous variables were summarized using median and interquartile range (IQR), and we also reported the prevalence of responses for each Likert scale category. Categorical variables were presented as frequencies and percentages. To ascertain the representativeness of our study sample, comparative analyses were conducted using a chi‐square test between the study participants and the entire Chinese population in terms of demographic characteristics, including age, gender, urban–rural distribution, and educational attainment. The statistics pertaining to the “total Chinese population” were sourced from the Seventh National Population Census of China in 2021 (OotLGotSCftSNP 2021). Importantly, as the primary aim of this study was to examine associations between symptoms rather than to produce nationally weighted prevalence estimates, post‐stratification weights were not applied in the network estimation or NCT analyses. Therefore, the findings should be interpreted within the present sample, and generalizability to the broader population warrants further validation.

#### 2.3.2 Network Estimation

Symptom networks were constructed using the R package qgraph (v1.9.2) with Graphical Gaussian Models (GGMs) (Epskamp et al. 2012). Nodes corresponded to individual items from the PHQ‐9 and GAD‐7 scales. Inspection of item‐level frequency distributions indicated uneven response distributions across several BMI categories (see Table 2), which may compromise the stability of polychoric correlation estimation. Given the non‐normality of the data and potential sparsity in ordinal response categories, edges were estimated using regularized Spearman partial correlations to obtain stable estimates of conditional associations among symptoms (Chen, Tang, et al. 2024; Epskamp et al. 2018). Edges represent conditional associations between symptoms after accounting for all other symptoms in the network. To obtain a sparse and interpretable network structure, we applied the graphical LASSO (least absolute shrinkage and selection operator) with the extended Bayesian information criterion (EBIC) and a tuning parameter *γ* = 0.5, which penalizes weak edges and retains only robust conditional associations (Epskamp et al. 2018). This regularization procedure reduces the risk of spurious connections and yields a parsimonious network representation.

Although PHQ‐9 and GAD‐7 items are ordinal, we encountered computational difficulties with polychoric correlations due to data sparsity in some subgroups. Nevertheless, to validate our methodological choice, we conducted sensitivity analyses using polychoric correlations with the same tuning parameter (*γ* = 0.5). These results are reported in Supporting Information A and are consistent with the primary findings.

#### 2.3.3 Network Centrality

To quantify node centrality, we used expected influence (EI). EI is the sum of all edge weights connected to a target node, preserving the sign of associations. This index is preferred over traditional centrality indices in networks that may contain negative edges (McNally 2016). Higher EI values indicate greater relative importance of a node within the network architecture, consistent with established network psychometric principles. This metric has demonstrated particular utility in identifying pivotal intervention targets for depression‐anxiety comorbidity (Epskamp et al. 2018; Fried et al. 2020; Yang et al. 2024; Zhang et al. 2024).

#### 2.3.4 Network Stability

The accuracy and stability of the observed network model were evaluated using the R‐bootnet (Version 1.5) package (Epskamp et al. 2018). Two primary analyses were conducted. The first assessed the stability of node centrality through a case‐drop bootstrap procedure with 1000 iterations, focusing on the EI index. The correlation stability coefficient (CS) was calculated, with values above 0.25 considered acceptable and those over 0.50 regarded as favorable with a maximum value of 0.99 (Epskamp et al. 2018; Yang et al. 2024). The second analysis estimated the 95% confidence intervals (CIs) of edge weights using a nonparametric bootstrap procedure, also comprising 1000 iterations. Wider CIs indicated decreased reliability in edge weight estimations, while narrower CIs suggested a more reliable network (Epskamp et al. 2018). Furthermore, bootstrapped difference tests were conducted to examine differences in network properties, including node EIs and edge weights. The statistical significance of differences between two edge weights or two node centrality indices was determined by evaluating the 95% CIs. These analyses provide information on the stability and accuracy of the estimated network.

#### 2.3.5 Network Comparison

The Network Comparison Test (NCT) was employed to evaluate differences in network structure and global strength between the various groups. Specifically, the NCT simultaneously tests (a) network structure invariance, (b) global strength invariance, and (c) differences in individual edge weights and node EIs between two networks using a permutation‐based procedure. To further explore the potential gender‐based differences, the initial sample was split into male and female groups. Furthermore, we conducted a comparative analysis of networks of individuals within the same gender across different BMI subgroups, as well as networks of individuals within the same BMI category across BMI subgroups.

To ensure the robustness of the NCT and to minimize errors from multiple comparisons, we performed 1000 permutations. All *p* values were adjusted using the Benjamini‐Hochberg false discovery rate (FDR) corrections (Eklund et al. 2016; van Borkulo et al. 2023). The adjustment was applied to all tested edges or node EIs. Consistent with the primary network estimation, the EBIC tuning parameter was set to *γ* = 0.5 for the NCT procedure. Besides *p* value, we also reported the *E* value, defined as the mean absolute difference in node's EI between two observed networks during permutations. The *E* value served as a descriptive effect‐size index, where larger values indicated more pronounced differences in symptom‐level associations. The M value represented the value of the maximum difference in edge weights, whereas the S value quantified the overall difference in network connectivity by summing differences across all edge weights.

#### 2.3.6 Sensitivity Analyses

To evaluate the robustness of the primary network estimation based on Spearman correlations and the robustness of the NCT results under unbalanced sample sizes, two complementary sensitivity analyses were conducted. First, networks were re‐estimated using polychoric correlations under the same regularization framework (EBICglasso, *γ* = 0.5), given their theoretical suitability for ordinal data. However, due to sparse endorsement in extreme response categories across several BMI × gender strata, the polychoric correlation matrices were occasionally unstable and required adjustments (e.g., enforcing positive definiteness), particularly during resampling procedures. Despite these computational challenges, the overall network structure and centrality patterns showed substantial consistency with the primary Spearman‐based results (see Supporting Information A).

Second, to address potential bias arising from unequal subgroup sizes in the NCT, a size‐matched subsampling procedure was performed. For each pairwise comparison, we drew random subsamples from the larger group to match the sample size of the smaller group and re‐estimated the networks prior to conducting NCT (1000 iterations). This procedure allowed us to assess whether the observed group differences were robust to sample size imbalance (see Supporting Information B). The results of these analyses were largely consistent with the original findings. Together, these sensitivity analyses support the robustness and stability of the primary results.

## 3 Results

### 3.1 Descriptive Statistics

A total of 9091 participants were included in this study, of whom 54.35% were female. This sampling framework approximated national demographic distributions based on available census indicators from the Seventh National Population Census of China (2021). There were no statistically significant differences between our study participants and the general Chinese population across key demographic factors, including age, gender, educational level, and urban/rural residence (*p* > 0.05). 1111 (12.22%) were assigned to the underweight group, 5632 (61.95%) as the normal weight group, 1975 (21.72%) as the overweight group, and 373 (4.10%) as the obesity group (Table 1). The sample sizes for gender‐stratified BMI subgroups were as follows: for males, underweight *n* = 310, normal weight *n* = 2445, overweight *n* = 1141, obesity *n* = 254; for females, underweight *n* = 801, normal weight *n* = 3187, overweight *n* = 834, obesity *n* = 119. Notably, the obesity group, particularly after gender stratification, had a relatively small sample size, which may affect the stability of subgroup‐specific network estimates.

**TABLE 1:** Descriptive statistics of participants (*n* = 9091).

Variables	Study participants	Total Chinese population (million)	*p*
Age, *n* (%)			0.213
19–25	2022 (22.24)	11,791 (12.60)	
26–35	2511 (27.62)	21,598 (23.07)	
36–45	2068 (22.75)	19,196 (20.51)	
46–65	2490 (27.39)	41,016 (43.82)	
Gender, *n* (%)			0.157
Male	4150 (45.65)	72,334 (51.24)	
Female	4941 (54.35)	68,844 (48.76)	
Educational level, *n* (%)			0.199
Primary school and below	588 (6.47)	38,741 (29.67)	
Junior high school and senior school	2505 (27.55)	70,017 (53.61)	
Junior college and above	5998 (65.98)	21,836 (16.72)	
Urban/rural distribution, *n* (%)			0.157
Urban	6773 (74.50)	90,199 (63.89)	
Rural	2318 (25.50)	50,979 (36.11)	
BMI (kg/m^2^), mean (SD)	22.07 (3.14)		
BMI categories, *n* (%)			
Underweight (BMI < 18.5)	1111 (12.22%)	—	
Normal weight (18.5 ≤ BMI < 24.0)	5632 (61.95%)	—	
Overweight (24.0 ≤ BMI < 28.0)	1975 (21.72%)	—	
Obesity (BMI ≥ 28.0)	373 (4.10%)	—	

A significant association was observed between BMI and the total score of depression (Table S1). This association appeared more pronounced in individuals categorized in the underweight and obesity groups. In the underweight group, the prevalence of moderate depression was 14.31%, the prevalence of severe depression was 12.06%, both significantly higher than those in the normal weight group (moderate depression: 9.75%; severe depression: 9.61%) and the overweight group (moderate depression: 9.52%; severe depression: 9.27%). Additionally, the prevalence of severe depression in the obesity group was 11.80%, which also exceeded that observed in the normal and overweight groups. In contrast, the differences in anxiety severity among the BMI groups were less pronounced (*p* = 0.090). The prevalence of severe anxiety in the underweight group was 4.05%, slightly higher than in the normal weight group (2.61%) and the overweight group (2.63%).

As illustrated in Tables S2 and S3, the distribution of depression and anxiety severity varied across BMI subgroups and genders. In both males and females, most participants in each BMI group exhibited no or mild depression and anxiety. However, higher proportions of moderate and severe depression were observed in the underweight and obesity groups for both genders. Notably, females in the underweight group reported more mild depression and anxiety compared to males, while males with obesity exhibited slightly higher levels of severe depression. For anxiety, severe cases remained relatively low across all groups, with slightly elevated rates among underweight participants. Chi‐square tests indicated significant differences in the distribution of depression severity across female BMI subgroups (*p* < 0.001) and anxiety severity across both males and females (*p* = 0.044 and *p* = 0.039, respectively). Table 2 presents the medians and interquartile ranges (IQRs; 25th–75th percentiles), proportions for each Likert‐scale response category, expected influence (EI) values, and predictabilities of all symptoms across BMI groups. PHQ‐9 and GAD‐7 items were scored on a 4‐point Likert scale (0–3), and EI values were standardized *Z* scores indicating the extent to which a node was influenced by other nodes.

**TABLE 2:** Descriptive statistics between different BMI subgroups based on PHQ9 and GAD7.

Group	Node	Item	Median (IQR)	Likert scale propotion				EI	Predictability
				0 (%)	1 (%)	2 (%)	3 (%)		
All (*n* = 9091)	PHQ1	Anhedonia	1 (0, 1)	36.1	48.1	12.2	3.6	0.90	0.63
	PHQ2	Sad mood	1 (0, 1)	48.0	39.5	10.1	2.5	0.96	0.68
	PHQ3	Sleep problems	1 (0, 1)	40.2	41.9	13.4	4.4	0.78	0.55
	PHQ4	Fatigue	1 (0,1)	32.9	50.1	12.8	4.3	0.97	0.64
	PHQ5	Appetite	1 (0, 1)	42.8	44.4	10.2	2.6	0.88	0.61
	PHQ6	Guilt	1 (0, 1)	49.3	36.7	11.0	3.0	0.99	0.68
	PHQ7	Concentration	1 (0,1)	47.4	38.3	11.2	3.1	0.92	0.63
	PHQ8	Motor problems	0 (0, 1)	58.1	29.3	10.1	2.4	1.02	0.69
	PHQ9	Suicide ideation	0 (0, 1)	74.5	15.4	7.9	2.3	0.76	0.61
	GAD1	Nervousness	1 (0, 1)	43.4	44.3	10.0	2.3	0.98	0.72
	GAD2	Uncontrollable worry	0 (0,1)	52.1	35.8	9.4	2.7	1.04	0.75
	GAD3	Excessive worry	1 (0, 1)	46.2	40.5	10.7	2.7	0.98	0.73
	GAD4	Trouble relaxing	1 (0, 1)	48.4	38.9	9.7	3.0	1.02	0.74
	GAD5	Restlessness	0 (0, 1)	55.4	33.5	9.1	2.0	1.01	0.75
	GAD6	Irritability	1 (0, 1)	46.5	41.3	9.9	2.4	0.95	0.71
	GAD7	Feeling afraid	0 (0, 1)	59.0	29.6	9.0	2.3	1.01	0.74
Underweight (*n* = 1111)	PHQ1	Anhedonia	1 (0, 1)	36.0	50.0	14.0	5.5	0.89	0.64
	PHQ2	Sad mood	1 (0, 1)	40.4	44.7	11.2	3.7	0.87	0.66
	PHQ3	Sleep problems	1 (0, 1)	37.3	41.4	15.5	5.9	0.76	0.54
	PHQ4	Fatigue	1 (0, 1)	29.0	51.0	14.3	5.7	0.87	0.64
	PHQ5	Appetite	1 (0, 1)	36.6	46.4	12.0	5.0	0.67	0.60
	PHQ6	Guilt	1 (0, 1)	43.0	39.8	12.7	4.5	0.95	0.66
	PHQ7	Concentration	1 (0, 1)	41.9	40.5	12.4	5.1	0.96	0.66
	PHQ8	Motor problems	0 (0, 1)	54.5	31.5	10.5	3.5	0.85	0.67
	PHQ9	Suicide ideation	0 (0, 1)	70.0	18.0	8.6	3.3	0.64	0.62
	GAD1	Nervousness	1 (0, 1)	37.3	48.3	11.0	3.4	0.88	0.73
	GAD2	Uncontrollable worry	1 (0, 1)	47.3	38.5	10.3	3.9	1.01	0.75
	GAD3	Excessive worry	1 (0, 1)	42.0	42.6	11.6	3.8	0.94	0.75
	GAD4	Trouble relaxing	1 (0,1)	45.9	40.6	9.9	3.6	0.96	0.76
	GAD5	Restlessness	0 (0, 1)	53.0	33.8	10.1	3.2	1.04	0.78
	GAD6	Irritability	1 (0, 1)	42.6	43.3	10.2	4.0	0.89	0.74
	GAD7	Feeling afraid	0 (0, 1)	54.4	32.8	9.7	3.2	1.02	0.77
Normal (*n* = 5632)	PHQ1	Anhedonia	1 (0, 1)	36.5	48.5	11.9	3.1	0.90	0.63
	PHQ2	Sad mood	1 (0, 1)	48.6	39.2	10.0	2.2	0.93	0.68
	PHQ3	Sleep problems	1 (0, 1)	41.2	41.6	13.3	3.9	0.78	0.56
	PHQ4	Fatigue	1 (0, 1)	33.8	49.7	12.7	3.8	0.95	0.64
	PHQ5	Appetite	1 (0, 1)	43.5	44.0	10.2	2.3	0.88	0.62
	PHQ6	Guilt	0 (0, 1)	50.0	36.2	11.2	2.6	1.00	0.69
	PHQ7	Concentration	1 (0, 1)	48.2	37.8	11.3	2.7	0.91	0.64
	PHQ8	Motor problems	0 (0, 1)	58.4	29.3	10.1	2.1	1.02	0.70
	PHQ9	Suicide ideation	0 (0, 1)	74.8	15.2	7.9	2.1	0.73	0.62
	GAD1	Nervousness	1 (0, 1)	43.4	44.2	10.2	2.1	0.98	0.72
	GAD2	Uncontrollable worry	0 (0, 1)	52.2	35.8	9.5	2.5	0.99	0.75
	GAD3	Excessive worry	1 (0, 1)	46.8	39.8	10.8	2.6	1.00	0.74
	GAD4	Trouble relaxing	1 (0, 1)	49.0	38.1	10.2	2.7	1.01	0.74
	GAD5	Restlessness	0 (0, 1)	55.6	33.1	9.5	1.8	0.99	0.75
	GAD6	Irritability	1 (0, 1)	47.4	40.6	10.0	2.1	0.94	0.72
	GAD7	Feeling afraid	0 (0, 1)	59.3	29.2	9.3	2.2	1.03	0.75
Overweight (*n* = 1975)	PHQ1	Anhedonia	1 (0, 1)	37.6	47.0	11.6	3.7	0.87	0.61
	PHQ2	Sad mood	0 (0, 1)	50.2	37.9	9.7	2.2	0.89	0.68
	PHQ3	Sleep problems	1 (0, 1)	38.9	43.7	12.6	4.8	0.80	0.53
	PHQ4	Fatigue	1 (0, 1)	32.5	50.8	12.3	4.4	1.03	0.63
	PHQ5	Appetite	1 (0, 1)	43.9	44.5	9.3	2.3	0.74	0.56
	PHQ6	Guilt	0 (0, 1)	50.5	37.3	9.2	3.0	0.98	0.67
	PHQ7	Concentration	1 (0, 1)	48.5	38.7	10.1	2.7	0.86	0.60
	PHQ8	Motor problems	0 (0, 1)	59.6	28.7	9.3	2.4	1.00	0.67
	PHQ9	Suicide ideation	0 (0, 0)	75.8	14.7	7.4	2.0	0.74	0.58
	GAD1	Nervousness	1 (0, 1)	46.4	42.7	8.8	2.1	0.98	0.72
	GAD2	Uncontrollable worry	0 (0, 1)	54.0	34.3	9.4	2.3	1.05	0.76
	GAD3	Excessive worry	1 (0, 1)	46.9	40.5	10.5	2.1	0.99	0.71
	GAD4	Trouble relaxing	1 (0, 1)	48.2	40.2	8.5	3.2	0.97	0.72
	GAD5	Restlessness	0 (0, 1)	56.1	34.3	7.9	1.7	1.06	0.73
	GAD6	Irritability	1 (0, 1)	46.1	42.2	9.6	2.2	0.88	0.68
	GAD7	Feeling afraid	0 (0, 1)	60.7	29.1	8.2	2.1	0.93	0.70
Obesity (*n* = 373)	PHQ1	Anhedonia	1 (0, 1)	38.9	42.9	13.7	4.6	0.86	0.70
	PHQ2	Sad mood	0 (0, 1)	50.1	36.5	9.1	4.3	0.59	0.72
	PHQ3	Sleep problems	1 (0, 1)	40.5	38.9	14.2	6.4	0.60	0.61
	PHQ4	Fatigue	1 (0, 1)	33.2	48.8	12.3	5.6	1.00	0.67
	PHQ5	Appetite	1 (0, 1)	44.2	42.6	9.7	3.5	0.71	0.68
	PHQ6	Guilt	0 (0, 1)	51.5	32.4	11.5	4.6	0.94	0.72
	PHQ7	Concentration	1 (0, 1)	45.6	36.5	11.8	6.2	0.70	0.65
	PHQ8	Motor problems	0 (0, 1)	57.4	26.8	12.6	3.2	0.96	0.72
	PHQ9	Suicide ideation	0 (0, 0)	75.1	13.9	7.8	3.2	0.64	0.65
	GAD1	Nervousness	1 (0, 1)	46.9	40.5	9.9	2.7	1.00	0.74
	GAD2	Uncontrollable worry	0 (0, 1)	54.4	35.9	6.2	3.5	0.84	0.76
	GAD3	Excessive worry	1 (0, 1)	45.8	43.4	7.2	3.5	0.63	0.75
	GAD4	Trouble relaxing	1 (0, 1)	48.3	39.4	8.6	3.8	1.04	0.77
	GAD5	Restlessness	0 (0, 1)	55.2	35.7	6.4	2.7	0.88	0.77
	GAD6	Irritability	1 (0, 1)	46.1	41.6	9.4	2.9	0.80	0.73
	GAD7	Feeling afraid	0 (0, 1)	59.0	30.0	7.8	3.2	0.70	0.74

### 3.2 Network Structure and Centrality

**FIGURE 1:**
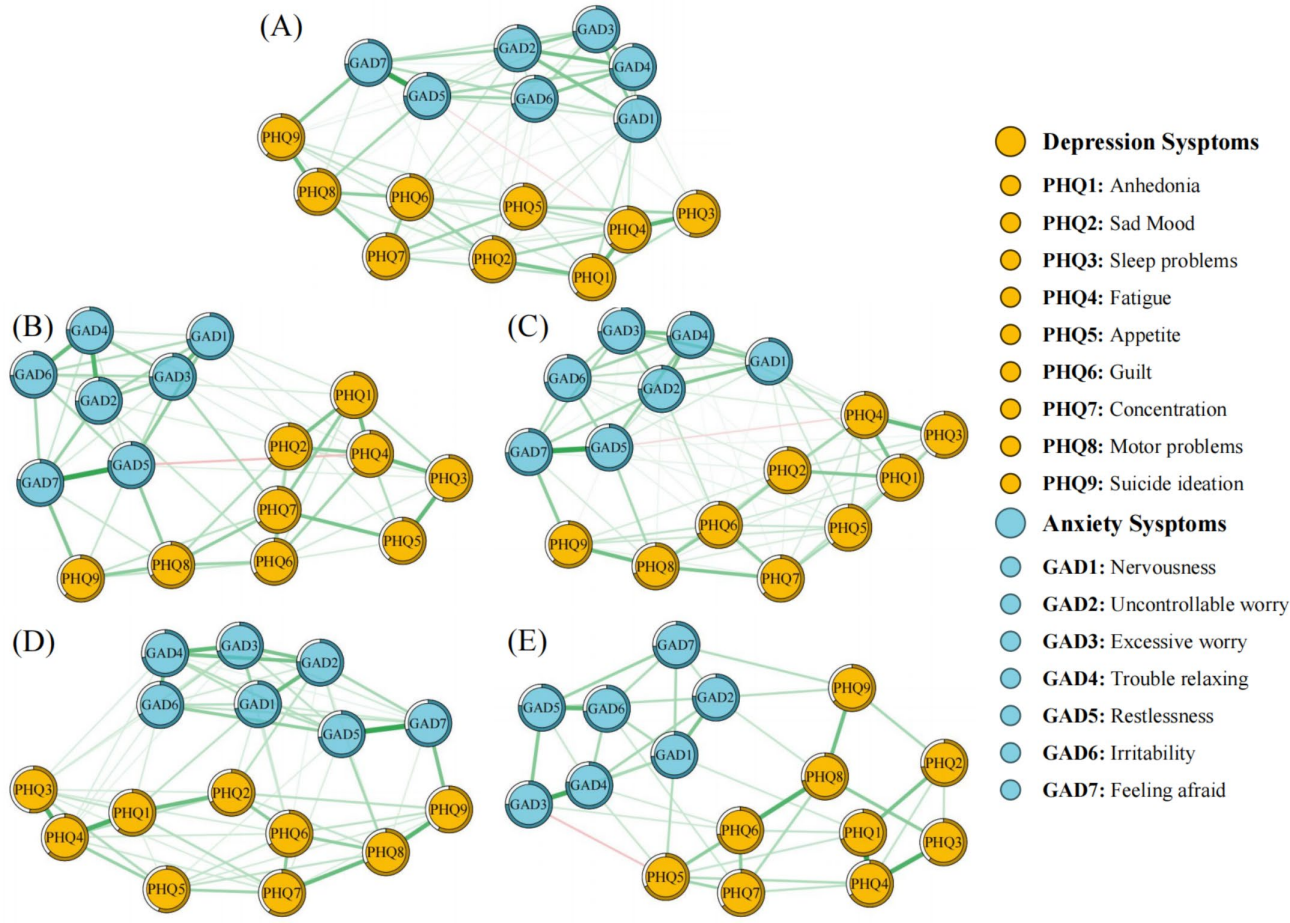
Depression–anxiety symptom network structures for different BMI subgroups. (A) Network of all Chinese adults. (B) Network of the underweight group. (C) Network of the normal weight group. (D) Network of the overweight group. (E) Network of the obesity group.

**FIGURE 2:**
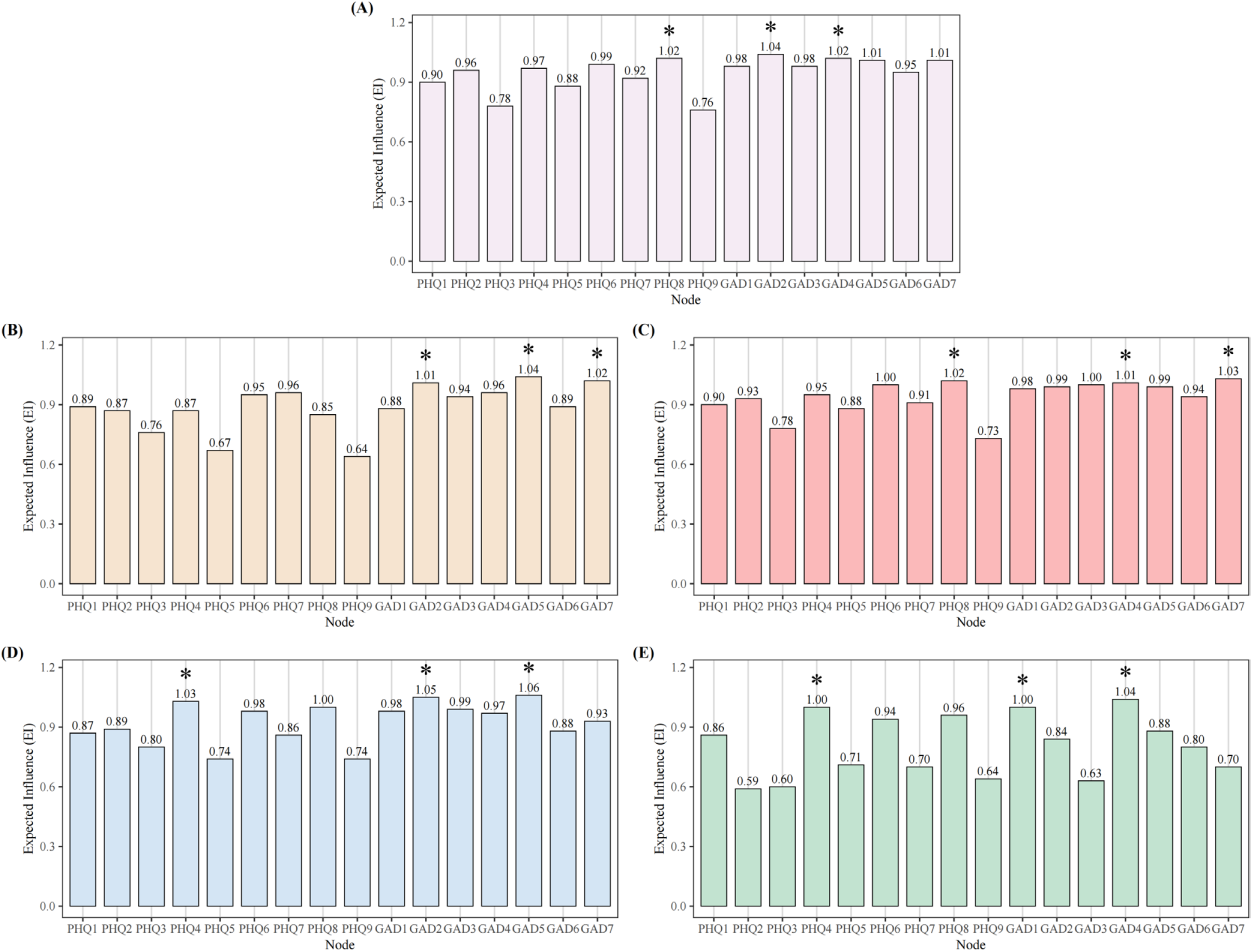
Expected Influence for different BMI subgroups' network. EI centrality values of PHQ‐9 and GAD‐7 symptoms in the overall sample (A), underweight subgroup (B), normal weight subgroup (C), overweight subgroup (D), and obesity subgroup (E). Bars represent EI values for each symptom. Asterisks (*) indicate the three symptoms with the highest EI values within each subgroup network.

Depression‐anxiety symptom networks were constructed for each of the four BMI categories and for the full sample, as shown in Figure 1A–E. Related network matrices are shown in Tables S4–S8. Node positioned closer together indicate stronger associations, with edge thickness indicating correlation strength (negative correlations are indicated by red lines, and positive correlations by green lines). The EI centrality of all symptom nodes in the full sample and each BMI subgroup is presented in Figure 2 and Table 2. In Figure 2, asterisks indicate the three symptoms with the highest EI values within each group, representing the core symptoms of the corresponding network. In addition, Table 3 summarizes the core symptoms and the strongest edges for the full sample and each BMI‐specific network.

**TABLE 3:** Core symptoms and the strongest edge of network in the full sample and different BMI subgroups.

Group	Core symptoms	The strongest edge
All	PHQ8, GAD2, GAD4	GAD5‐GAD7
Underweight	GAD2, GAD5, GAD7	GAD5‐GAD7
Normal	PHQ8, GAD4, GAD7	GAD5‐GAD7
Overweight	PHQ4, GAD2, GAD5	GAD5‐GAD7
Obesity	PHQ4, GAD1, GAD4	PHQ1‐PHQ4

In the network for all participants (Figure 1A), 72 edges (52.94%) out of 136 possible edges were retained as having nonzero values, with one edge showing a negative weight. The strongest edge was between the symptoms of “Restlessness” (GAD5) and “Feeling afraid” (GAD7). Notably, “Uncontrollable worry” (GAD2), “Motor problems” (PHQ8) and “Trouble relaxing” (GAD4) emerged as the nodes with the highest EI values, as detailed in Table 2 and Figure 2. Furthermore, the mean predictability across all nodes was determined to be 0.68 ± 0.06, suggesting that the connections from adjacent nodes could account for 68% of the overall variance.

For the underweight group (Figure 1B), a total of 69 positive edges and 1 negative edge were detected, constituting 51.47% of all possible edges. The strongest edge again connected “restlessness” and “feeling afraid” (GAD5‐GAD7). As shown in Table 2 and Figure 2, “restlessness” (GAD5), “feeling afraid” (GAD7) and “uncontrollable worry” (GAD2) showed the highest node EI values. On average, neighboring nodes explained 69% of node variance on average (*M*_predictability_ = 0.69 ± 0.07).

For participants classified as the normal weight group (Figure 1C), 71 edges were retained, representing 52.21% of the potential edges, with one negative weight edge. The strongest association remained between “restlessness” and “feeling afraid” (GAD5‐GAD7). Table 2 and Figure 2 indicated that “feeling afraid” (GAD7), “motor problems” (PHQ8) and “trouble relaxing” (GAD4) exhibited the highest EI values. The mean predictability indicated that approximately 68% of the variance of each node could be explained by its neighboring nodes (*M*_*predictability*_ = 0.68 ± 0.06).

In the overweight group (Figure 1D), 70 positive edges were identified, amounting to 51.47% of the potential edges, with no negative edges present. The strongest correlation was again between “restlessness” (GAD5) and “feeling afraid” (GAD7). According to Table 2 and Figure 2, “restlessness” (GAD5), “uncontrollable worry” (GAD2) and “fatigue” (PHQ4) exhibited the highest EI values. On average, 66% of the network variance was attributable to the influence of neighboring nodes (*M*_predictability_ = 0.66 ± 0.07).

For the obesity group (Figure 1E), 60 positive edges and 1 negative edge were found (44.85%) out of the 136 possible edges. The strongest association was discovered between “anhedonia” (PHQ1) and “fatigue” (PHQ4), with a single edge displaying a negative weight (PHQ5‐GAD3). Table 2 and Figure 2 indicated that “trouble relaxing” (GAD4), “nervousness” (GAD1) and “fatigue” (PHQ4) possessed the highest EI values. Notably, the average prevalence of variance explained by neighboring nodes in the network was approximately 71% (*M*_predictability_ = 0.71 ± 0.05). However, these findings should be interpreted with caution given the relatively smaller sample size of the obesity subgroup.

Sensitivity analyses using polychoric correlations yielded highly similar network structures and centrality rankings (see Supporting Information A), supporting the robustness of our primary Spearman‐based networks. Moreover, the subsampling NCT results were consistent with the main NCT findings (see Supporting Information B), indicating that sample size imbalances did not substantially bias the comparison results.

### 3.3 Network Accuracy and Stability

The depression‐anxiety networks for each BMI group exhibited high stability and accuracy after 1000 bootstrap iterations. The narrow 95% confidence intervals indicated that the accuracy of the symptom network (Figure S1) was consistently reliable across all groups. Furthermore, the CS‐coefficients for node EI all exceeded 0.25, indicating that the EI values remained highly correlated with those from the original data after dropping up to 25% of the sample (Table S9). Moreover, the 95% bootstrapped confidence intervals of the edge weights were also narrow (Figure S2), indicating that the network edges were robust. Additionally, the nonparametric bootstrapping analysis revealed statistically significant differences in most comparisons between edge weights and EI centrality indicators, as illustrated in Figures S3 and S4.

### 3.4 Network Comparison Test of BMI Subgroups and Gender‐Specific Networks

**FIGURE 3:**
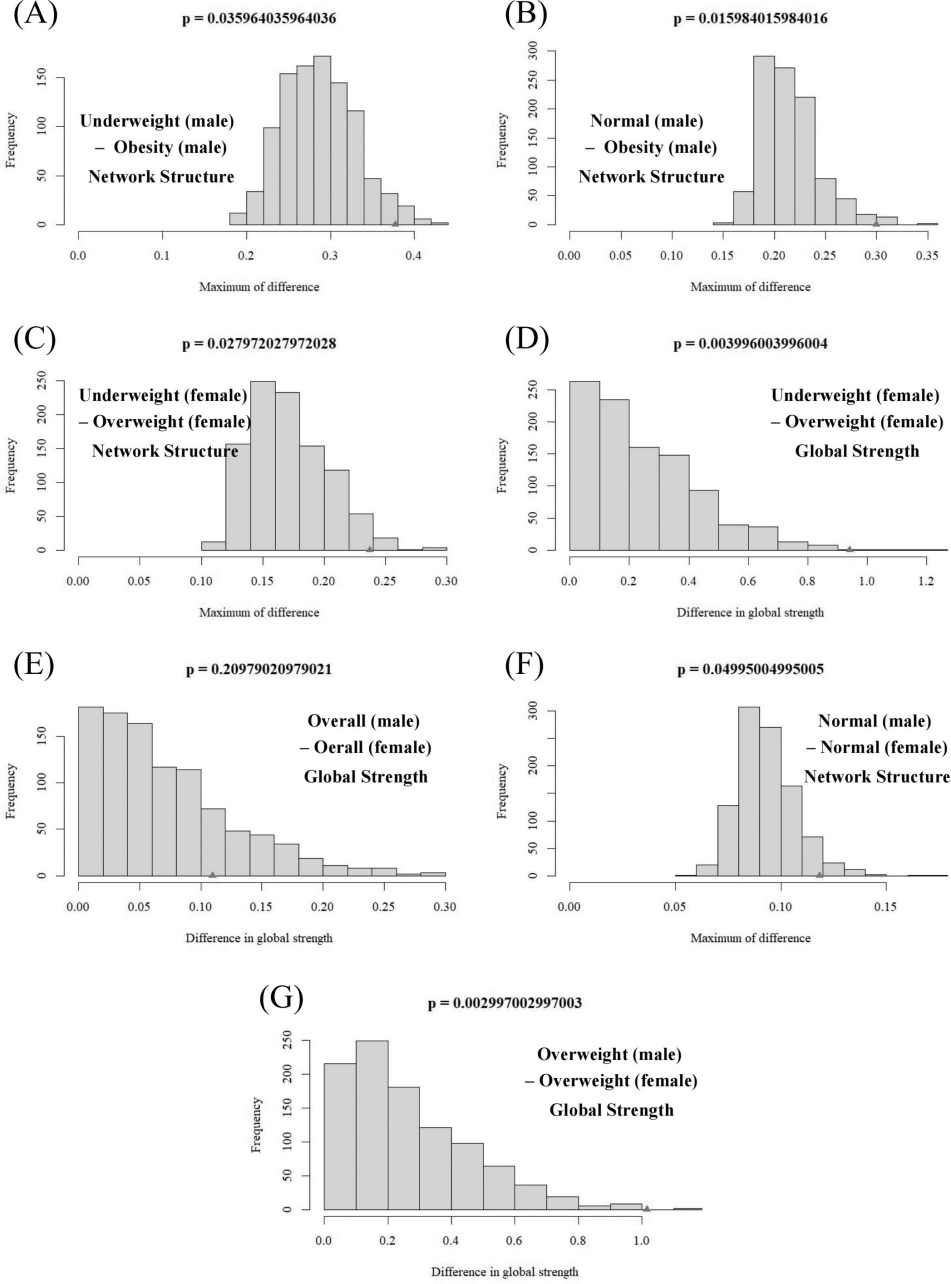
Network comparison results of BMI subgroups and gender‐specific networks. (A) The network structure invariance results between underweight and obesity group in male. (B) The network structure invariance results between normal and obesity group in male. (C) The network structure invariance results between underweight and overweight group in female. (D) The global strength invariance results between underweight and overweight group in female. (E) The global strength invariance results between male and female in the full sample. (F) The network structure invariance results between male and female in the normal group. (G) The global strength invariance results between male and female in the overweight group.

Figure 3 illustrates the significant differences in NCT across BMI subgroups and gender‐specific networks. As shown in Table 4, no significant differences were found in network structure or global strength across BMI subgroups. Further gender‐specific analysis revealed distinctions. For males (see Table 8), a difference in the EI of the node “feeling afraid” (GAD7) was observed between the normal and obesity groups (*p* = 0.040, *E* = 0.450). In Table 5, a significant structural difference was observed between the underweight and obesity groups (*p* = 0.036) and between the normal and obesity groups (*p* = 0.016). For females (see Table 6), only the comparison between the underweight and overweight groups showed significant differences in both network structure (*p* = 0.028) and global strength (*p* = 0.004).

**TABLE 4:** Network comparison results between different BMI subgroups.

	Network structure invariance	Global strength invariance
Underweight–normal	*M* = 0.125 *p* = 0.253	Underweight = 7.098, normal = 7.513 *S* = 0.414 *p* = 0.750
Underweight–overweight	*M* = 0.125 *p* = 0.568	Underweight = 7.098, overweight = 7.375 *S* = 0.277 *p* = 0.488
Underweight – Obesity	*M* = 0.230 *p* = 0.242	Underweight = 7.098, obesity = 6.447 *S* = 0.652 *p* = 0.502
Normal–overweight	*M* = 0.099 *p* = 0.221	Normal = 7.513, overweight = 7.375 *S* = 0.138 *p* = 0.841
Normal–obesity	*M* = 0.184 *p* = 0.452	Normal = 7.513, obesity = 6.447 *S* = 1.066 *p* = 0.558
Overweight–obesity	*M* = 0.194 *p* = 0.473	Overweight = 7.375, obesity = 6.447 *S* = 0.929 *p* = 0.621

**TABLE 5:** Network comparison results between male subjects across different BMI subgroups.

	Network structure invariance	Global strength invariance
Underweight (male)–normal (male)	*M* = 0.175 *p* = 0.859	Underweight = 6.658, normal = 7.415 *S* = 0.757 *p* = 0.486
Underweight (male)–overweight (male)	*M* = 0.218 *p* = 0.514	Underweight = 6.658, overweight = 7.282 *S* = 0.624 *p* = 0.513
Underweight (male)–obesity (male)	*M* = 0.377 ***p* = 0.036**	Underweight = 6.658, obesity = 6.101 *S* = 0.557 *p* = 0.362
Normal (male)–overweight (male)	*M* = 0.145 *p* = 0.150	Normal = 7.415, overweight = 7.282 *S* = 0.134 *p* = 0.613
Normal (male)–obesity (male)	*M* = 0.300 ***p* = 0.016**	Normal = 7.415, obesity = 6.101 *S* = 1.314 *p* = 0.193
Overweight (male)–obesity (male)	*M* = 0.275 *p* = 0.168	Overweight = 7.282, obesity = 6.101 *S* = 1.180 *p* = 0.272

*Note:* the bold values highlights the comparison results between groups which are statistic significant differences.

**TABLE 6:** Network comparison results between female subjects across Different BMI subgroups.

	Network structure invariance	Global strength invariance
Underweight (female)–normal (female)	*M* = 0.154 *p* = 0.254	Underweight = 7.098, normal = 7.318 *S* = 0.111 *p* = 0.996
Underweight (female)–overweight (female)	*M* = 0.237 ***p* = 0.028**	Underweight = 7.208, overweight = 6.267 *S* = 0.941 ***p* = 0.004**
Underweight (female)–obesity (female)	*M* = 0.311 *p* = 0.347	Underweight = 7.208, obesity = 6.501 *S* = 0.707 *p* = 0.635
Normal (female)–overweight (female)	*M* = 0.141 *p* = 0.332	Normal = 7.318, overweight = 6.267 *S* = 1.051 *p* = 0.106
Normal (female)–obesity (female)	*M* = 0.311 *p* = 0.241	Normal = 7.318, obesity = 6.501 *S* = 0.818 *p* = 0.890
Overweight (female)–obesity (female)	*M* = 0.292 *p* = 0.694	Overweight = 6.267, obesity = 6.501 *S* = 0.234 *p* = 0.893

*Note:* the bold values highlights the comparison results between groups which are statistic significant differences.

Comparisons between males and females within the same BMI category are detailed in Table 7. For the normal BMI group, a subtle but statistically significant variation in the network structure was found (*p* = 0.050). The overall sample (*p* = 0.003) and the overweight group (*p* = 0.001) exhibited pronounced differences in global strength. There were no significant gender differences in edge weights or node EIs within the underweight, normal weight, or obesity groups. For the overall and overweight groups, while no significant differences were observed in the edges, notable distinctions were identified at the node level. Significant differences were detected among the following nodes (see Table 8): Overall group: PHQ5 (*p* = 0.032, *E* = 0.073); Overweight group: PHQ3 (*p* = 0.027, *E* = 0.359), PHQ5 (*p* = 0.016, *E* = 0.476), PHQ6 (*p* = 0.032, *E* = 0.337), and PHQ9 (*p* = 0.027, *E* = 0.318). For these nodes, males exhibited higher EI values compared with females.

**TABLE 7:** Network comparison results between male and female subjects in the full sample or BMI subgroups.

	Network structure invariance	Global strength invariance
All (male)–all (female)	*M* = 0.074 *p* = 0.243	Male = 7.692, female = 7.602 *S* = 0.090 ***p* = 0.001**
Underweight (male)–underweight (female)	*M* = 0.205 *p* = 0.741	Male = 6.658, female = 7.208 *S* = 0.549 *p* = 0.465
Normal (male)–normal (female)	*M* = 0.118 ***p* = 0.050**	Male = 7.415, female = 7.318 *S* = 0.097 *p* = 0.499
Overweight (male)–overweight (female)	*M* = 0.154 *p* = 0.566	Male = 7.282, female = 6.267 *S* = 1.015 ***p* = 0.003**
Obesity (male)–obesity (female)	*M* = 0.353 *p* = 0.486	Male = 6.101, female =6.501 *S* = 0.399 *p* = 0.619

*Note:* the bold values highlights the comparison results between groups which are statistic significant differences.

**TABLE 8:** Difference in individual node EIs between male and female subjects across BMI subgroups by NCT.

Node	Group1	Group2	Difference significance
GAD7	Normal (male): 1.007	Obesity (male): 0.557	*p* = 0.040, *E* = 0.450
PHQ5	Overall (male): 0.871	Overall (female): 0.820	*p* = 0.032, *E* = 0.073
PHQ3	Overweight (male): 0.712	Overweight (female): 0.353	*p* = 0.027, *E* = 0.359
PHQ5	Overweight (male): 0.849	Overweight (female): 0.373	*p* = 0.016, *E* = 0.476
PHQ6	Overweight (male): 1.025	Overweight (female): 0.689	*p* = 0.032, *E* = 0.337
PHQ9	Overweight (male): 0.753	Overweight (female): 0.435	*p* = 0.027, *E* = 0.318

To address the potential impact of unbalanced samples in NCT, we conducted a sensitivity analysis using size‐matched subsampling. Across 1000 iterations, the subsampling analysis yielded results that were largely consistent with the primary findings, with similar patterns of network structure and global strength differences observed between groups (see Tables S10–S13). These results indicate that the identified group differences are unlikely to be driven by sample size imbalance. Overall, the consistency of findings across multiple analytical approaches supports the robustness of the reported network characteristics.

## 4 Discussion

The present study examined depression‐anxiety symptom networks across BMI and gender subgroups in Chinese adults. In the overall sample, “uncontrollable worry” (GAD2), “motor problems” (PHQ8) and “trouble relaxing” (GAD4) emerged as the most central symptoms. Core symptoms varied across BMI subgroups, and higher levels of depressive symptoms were observed in the underweight and obesity groups. Although no substantial differences were found across BMI groups in the overall network comparisons, additional differences emerged after gender stratification. Among males, differences in network structure were mainly observed in comparisons involving the obesity group. Females exhibited differences in network structure and global strength for the underweight and overweight groups. Male–female differences were also identified in global strength for the overall sample and the overweight group, and network structure also differed within the normal weight group. Overall, these findings provide a nuanced view of how BMI and gender may be associated with depression‐anxiety symptom networks within the present sample.

### 4.1 Core Symptoms of the Depression‐Anxiety Symptom Network Across BMI Categories

The high EI values of “uncontrollable worry” (GAD2), “motor problems” (PHQ8) and “trouble relaxing” (GAD4) suggest that these symptoms may occupy relatively important positions in the depression‐anxiety symptom network across all participants. This pattern is broadly consistent with previous network studies in Chinese populations, which have also highlighted trouble relaxing and motor problems as prominent symptoms (Bai et al. 2022). One possible interpretation is that these symptoms reflect common manifestations of sustained psychological strain in adult daily life (Kivimäki and Steptoe 2018; Leif et al. 2018), although the cross‐sectional design does not allow conclusions about causal mechanisms. These findings may underscore the importance of emotional regulation in the context of chronic stress (Kivimäki and Steptoe 2018).

Among underweight participants, the prominence of anxiety‐related symptoms may be related to the co‐occurrence of anorexia nervosa (Calugi et al. 2013), nutritional vulnerability, fatigue, and psychological distress reported in previous studies (Inge and Ingela Lundin 2015; Thompson and Stice 2001). These factors may negatively affect neurotransmitter systems, such as decreased levels of serotonin, further aggravating symptoms of anxiety and depression (Ehrlich et al. 2010; Jonathan et al. 2011; Mondoloni et al. 2024). However, this interpretation remains speculative, and the present data do not allow direct examination of the biological mechanisms involved.

In the normal weight group, the central symptoms may reflect more general forms of psychological strain that are not specific to weight status, such as work‐related stress, uncertainty, and everyday emotional burden (Cathomas et al. 2024; Jürgen and Kevin D 2019). This interpretation is consistent with the view that distress networks in community adults may be shaped by multiple social and contextual factors beyond BMI alone (Yu et al. 2022).

In contrast, overweight and obese individuals face additional physiological and psychological burdens. Prior research has linked higher BMI to inflammation (Owein and Giulio G 2017; Sutin et al. 2014), fatigue (Wang, He, et al. 2024; Xia et al. 2021), chronic disease burden (e.g., type 2 diabetes, cardiovascular disease, and cancer) (Okunogbe et al. 2021; Riquelme et al. 2021), and weight‐related stigma, all of which may contribute to emotional distress (Braden et al. 2023; Chekroud et al. 2018). Interventions addressing the social stigma of body weight may be essential to improving mental health outcomes in this population (Duan and Feng 2018). At the same time, these mechanisms were not directly assessed in the present study and should therefore be interpreted with caution.

### 4.2 Interaction Between BMI and Gender in Relation to Depression and Anxiety

The underweight and obesity groups exhibited higher rates of depression than the normal weight and overweight groups. These findings are broadly consistent with prior literature describing a U‐shaped association between BMI and depression (He et al. 2022; Ma et al. 2021; Yeom and Kim 2022). Despite the absence of significant differences in network structure, global strength, and node centrality (EI) across the BMI subgroups, the chi‐squared test results suggested a different pattern across genders. These findings imply that BMI may not be the sole determinant of the network structure of depression‐anxiety symptoms. Consistent with prior research (Jung et al. 2023; McCrea et al. 2012; Park 2009; Ul‐Haq et al. 2014), the present results suggest that some aspects of network structure, global strength, and node centrality (EI) may vary by gender, underscoring the potential value of considering gender differences in future research and clinical interventions for adult mental health.

Across the full sample, males exhibited higher global strength and higher EI values for “Appetite” (PHQ5) compared to females. This may be related to gender differences in physiological responses to stress and divergent social expectations, although these factors were not directly measured in the present study. While females are often subject to cultural pressures to maintain slim physiques and fulfill traditional family roles, males face societal expectations to exhibit strength, self‐esteem, and responsibility (Gualdi‐Russo et al. 2022; Jo et al. 2024). These pressures may prompt males to suppress emotional distress and adopt maladaptive coping strategies, such as overeating (Du et al. 2022; Gualdi‐Russo et al. 2022; Wang et al. 2022). In the Chinese sociocultural context, body weight is not merely an individual attribute but is embedded within gendered role expectations and family‐oriented values, which may differentially shape how psychological distress is experienced and expressed by men and women.

In the normal weight group, although a subtle difference in network structure was observed between males and females (Li et al. 2023), no significant discrepancies were found in EI values or edge weights. This may reflect that people with normal BMI share similar coping strategies in response to emotional challenges, likely due to comparable levels of social acceptance and reduced weight‐related stigma, which diminishes gender‐based psychological differences, although this interpretation requires further empirical validation.

Gender differences were most pronounced in the overweight group. Here, males displayed significantly higher global network strength in depressive symptoms compared with females. However, anxiety‐related symptoms did not show significant gender‐specific variation. These findings align with previous research suggesting that overweight males may be more susceptible to depression than overweight females, despite both genders experiencing elevated risks of psychiatric disorders (Amiri and Behnezhad 2019; McCrea et al. 2012; Xie et al. 2023). From a cultural perspective, overweight status in men may subtly conflict with contemporary expectations of self‐discipline and competitiveness, potentially altering symptom connectivity, whereas women may internalize body dissatisfaction more directly, leading to heightened symptom severity, although this sociocultural explanation remains tentative.

Notably, no gender differences were observed in the underweight or obesity groups. This null finding warrants careful interpretation and may reflect several convergent processes. One possible explanation is a “stigma saturation” effect, whereby extreme deviations from normative body weight in the Chinese context carry such pervasive social penalties that gender‐specific experiences become less distinguishable. Underweight individuals may be perceived as frail or incapable of fulfilling expected family roles, whereas obese individuals may face strong social criticism in a culture that increasingly emphasizes self‐discipline and body management. Such shared social pressures may produce a convergence of psychological experiences across genders.

Physiological mechanisms may also contribute to this convergence. At weight extremes, nutritional deficiencies or metabolic disturbances may exert more direct effects on mood regulation and fatigue, potentially overshadowing the gender‐specific coping styles observed in other BMI groups. These biological stressors may act as common pathways that diminish the relative influence of gender on symptom presentation.

Additionally, methodological factors should be considered. The relatively smaller sample sizes in the underweight and particularly the obesity groups, especially after gender stratification, may have reduced statistical power to detect subtle differences in network parameters. Therefore, the absence of gender differences in these groups should be interpreted as a plausible pattern rather than definitive evidence of equivalence.

We also investigated the network differences between BMI subgroups within the same gender. For males, differences in symptom network structure were mainly observed in comparisons involving the obesity groups (underweight vs. obesity; normal weight vs. obesity). As traditional chi‐square analyses suggested associations between BMI and anxiety, but not depression, our findings suggest that BMI may be associated with psychological distress in males through changes in symptom connectivity by altering interconnections between symptoms rather than by directly increasing depressive symptom severity. This indicates that BMI's impact on males is more complex, manifesting through changes in symptom connectivity rather than simply increasing overall depression symptom severity.

For females, a different pattern emerged. Network differences were found between the underweight and overweight groups, while traditional chi‐square results suggested higher risks of depression and anxiety in these groups. These findings indicate that for females, BMI is associated not only with altered symptom networks but also with increased psychological burden—reflecting a stronger and more direct impact of BMI on mental health. Taken together, these results highlight that the interaction between BMI and gender is not uniform across the weight spectrum but is shaped by culturally embedded gender norms, social evaluation processes, and potentially shared biological stressors at weight extremes. These patterns underscore the importance of considering both gender and body weight as intersecting factors that jointly influence the presentation and connectivity of depressive and anxiety symptoms.

### 4.3 Potential Clinical Implications

The following clinical implications should be interpreted as hypothesis‐generating rather than prescriptive, given the cross‐sectional design and the methodological considerations noted above. In clinical practice, interventions for males could focus on highly connected symptoms within the network, particularly in individuals with extreme BMI statuses such as underweight or obesity. Our findings suggest that in males, BMI may interact with mental health more through alterations in symptom connectivity rather than through marked increases in overall symptom severity. If replicated, this pattern could suggest that screening efforts should pay particular attention to highly connected symptoms, such as appetite‐related changes and fatigue, especially in overweight or obese men, which may serve as potential entry points into broader depressive‐anxiety networks. Interventions for males may benefit from addressing core symptoms such as low mood, sleep disturbances, and physical fatigue while incorporating dietary and mood management support (Ramos‐Vera et al. 2022; Wang, Wu, et al. 2024; Xie et al. 2023). Additionally, it may be valuable to help males develop healthy coping strategies to reduce reliance on maladaptive behaviors like overeating (Wang, Wu, et al. 2024).

For females, interventions could focus on alleviating the severity of symptoms and mitigating symptom reinforcement effects to reduce the overall psychological burden. Given that BMI in females was associated not only with network alterations but also with elevated symptom severity in specific BMI groups, risk stratification may be particularly important for underweight and overweight women. Routine screening in these groups may prioritize mood‐related and self‐evaluative symptoms, which could signal heightened vulnerability. For underweight females, this may involve nutritional support combined with mood management strategies (Tang et al. 2024; Wang, He, et al. 2024; Wang, Wu, et al. 2024). For overweight females, psychological support aimed at enhancing self‐esteem and addressing body image concerns would be beneficial (Duan and Feng 2018). In such cases, integrated approaches that address both physiological regulation and internalized weight stigma may be more effective than symptom‐specific treatment alone.

Importantly, the absence of pronounced gender differences in the underweight and obesity groups suggests that, at BMI extremes, interventions may require a more unified framework that simultaneously addresses biological stressors and shared experiences of stigma, rather than highly gender‐differentiated strategies. However, these clinical interpretations should be considered hypothesis‐generating rather than prescriptive, as the cross‐sectional nature of the data limits causal inference and definitive conclusions about intervention sequencing cannot be drawn, particularly given the relatively small subgroup sizes after stratification.

### 4.4 Limitations and Future Directions

Several limitations should be considered. First, although the sampling strategy was designed to approximate national demographic distributions, the use of quota sampling without post‐stratification weighting may limit the representativeness of the sample. Therefore, the findings should be interpreted with caution and primarily understood within the context of the present sample. Meanwhile, we cannot entirely rule out residual confounding from unmeasured variables that were not included as nodes in the network. This is an inherent limitation of the current analytical approach. Future studies could consider more comprehensive confounder adjustment to further validate the robustness of the observed symptom networks. Second, the cross‐sectional design restricts the ability to draw causal inferences, and longitudinal studies are recommended to explore the dynamic relationship between BMI and mental health. Third, the reliance on self‐report measures (particularly height and weight) may be subject to systematic reporting biases. Despite the implementation of quality control procedures, biases such as social desirability and recall bias cannot be entirely ruled out, which could affect subgroup classification and, consequently, the estimated network structures. Future research could incorporate objective assessment to enhance accuracy and validate self‐reported data. Fourth, the relatively small sample sizes in certain subgroups, particularly the obesity group after gender stratification, may have reduced statistical power and affected the stability of subgroup‐specific network estimates. Although the sensitivity analyses provided some support for the robustness of the findings, they do not eliminate concerns related to representativeness, measurement error, or limited power in smaller subgroups.

## 5 Conclusion

The findings illustrate the intricate nature of the depression‐anxiety symptom networks across different BMI and gender groups. For males, BMI appeared to be associated more strongly with symptom connectivity, whereas in females, it appeared to be associated with both symptom severity and network characteristics in some BMI groups. These findings provide preliminary evidence for gender‐ and BMI‐related differences in depression‐anxiety symptom networks as well as potential clinical implications. Males may require support in managing symptom connectivity and unhealthy coping strategies, whereas females may benefit more from symptom relief and targeted nutritional or psychological support. However, given the methodological limitations, the results should be interpreted cautiously and require replication in future studies using more representative and longitudinal data.

## Funding

This work was supported by National Natural Science Foundation of China (72171193, 32500978, 72231008, 72271126), Science and Technology Innovation Team of Shaanxi Provincial (2024Rs‐CXTD‐28), Practice and Innovation Funds for Graduate Students of Northwestern Polytechnical University (PF2025049), 2025 Project of Shaanxi Provincial Sports Bureau (20250287), China Education Development Foundation, National Center for Mental Health and Mental Health, China; 'Sunflower Action for Promoting Students' Mental Health' of the Center for Student Services and Development; and the Ministry of Education, P.R. China.

## Ethics Statement

The studies involving human participants were reviewed and approved by Shaanxi Health Culture Research Centre (JKWH‐2021‐01) and the Ethics Committee of Jinan University (JNUKY‐2021‐018). Written informed consent was obtained from all participants prior to participation.

## Conflicts of Interest

The authors declare no conflicts of interest.

## Supporting information


**Figure S1:** The accuracy of the symptom networks' EI. The *x*‐axis indicates the percentage of cases of the original sample included at each step. The *y*‐axis indicates the average of correlations between the centrality indices from the original network and the centrality indices from the networks that were re‐estimated after excluding increasing percentages of cases. (A–C) indicates all Chinese adults, adult males, and adult females in aged 19–65. (D–F) indicates underweight group and its male and female subgroups. (G–I) indicates normal weight group and its male and female subgroups. (J–L) indicates overweight group and its male and female subgroups. (M–O) indicates obesity group and its male and female subgroups. The relatively narrow 95% confidence intervals indicated that the accuracy of the symptom networks were good for all groups.
**Figure S2:** Nonparametric bootstrapped confidence intervals of estimated edges in symptom networks. The red line represents the estimated edge, while the dark area indicates the 95% bootstrap confidence interval. (A–C) indicates all Chinese adults, adult males, and adult females in aged 19–65. (D–F) indicates underweight group and its male and female subgroups. (G–I) indicates normal weight group and its male and female subgroups. (J–L) indicates overweight group and its male and female subgroups. (M–O) indicates obesity group and its male and female subgroups. Edges in the networks were robust and can be trusted.
**Figure S3:** Bootstrapped stability test for edge‐weight in symptom networks. The results of the bootstrapped difference tests (*α* = 0.05) for edge‐weights were shown in this figure. The color of the boxes indicates whether edge‐weights differ significantly from each other (i.e., black) or do not differ significantly (i.e., gray). The diagonal line indicates the strength of edge‐weights, shifting from red (negative associations) to white (representing weaker edges) and ultimately blue (representing stronger edge‐weights). (A–C) indicates all Chinese adults, adult males, and adult females in aged 19 ~ 65. (D–F) indicates underweight group and its male and female subgroups. (G–I) indicates normal weight group and its male and female subgroups. (J–L) indicates overweight group and its male and female subgroups. (M–O) indicates obesity group and its male and female subgroups.
**Figure S4:** Nonparametric bootstrapped difference test for nodes in symptom networks. Gray boxes indicate no significant difference, whereas black boxes indicate a statistically significant difference (*p* < 0.05). Diagonal color and saturation represent the magnitude and direction of each estimated edge. (A–C) indicates all Chinese adults, adult males, and adult females in aged 19–65. (D–F) indicates underweight group and its male and female subgroups. (G–I) indicates normal weight group and its male and female subgroups. (J–L) indicates overweight group and its male and female subgroups. (M–O) indicates obesity group and its male and female subgroups.
**Table S1:** Distribution of BMI subgroups and associated levels of depression and anxiety (overall).
**Table S2:** Distribution of BMI subgroups and associated levels of depression and anxiety (male).
**Table S3:** Distribution of BMI subgroups and associated levels of depression and anxiety (female).
**Table S4:**. Weighted adjacency matrix of all participants.
**Table S5:**. Weighted adjacency matrix of underweight participants.
**Table S6:**. Weighted adjacency matrix of normal weight participants.
**Table S7:**. Weighted adjacency matrix of overweight participants.
**Table S8:**. Weighted adjacency matrix of obese participants.
**Table S9:** Node centrality's stability of network in the full sample and different BMI subgroups.
**Table S10:**. Network comparison results between different BMI subgroups using subsampling data.
**Table S11:**. Network comparison results between male subjects across Different BMI subgroups using subsampling data.
**Table S12:**. Network comparison results between female subjects across Different BMI subgroups using subsampling data.
**Table S13:**. Network comparison results between male and female subjects in the full sample or BMI subgroups using subsampling data.

## Data Availability

Our study was based on the dataset from the Psychology and Behavior Investigation of Chinese Residents (PBICR 2021), and access to the dataset was obtained legally. Data are available upon reasonable request and subject to approval and relevant restrictions.
