# Supplementary material for: Gender‐Specific Depression‐Anxiety Symptom Networks and the Impact of Weight Status: Insights From a Large‐Scale Study
Source: Psych J. 2026 May 29;15(3):e70104. doi: 10.1002/pchj.70104 (PMC13240150; doi:10.1002/pchj.70104)
Supplement: Supplementary file 1 — Figure S1: The accuracy of the symptom networks' EI. The x‐axis indicates the percentage of cases of the original sample included at each step. The y‐axis indicates the average of correlations between the centrality indices from the original network and the centrality indices from the networks that were re‐estimated after excluding increasing percentages of cases. (A–C) indicates all Chinese adults, adult males, and adult females in aged 19–65. (D–F) indicates underweight group and its male and female subgroups. (G–I) indicates normal weight group and its male and female subgroups. (J–L) indicates overweight group and its male and female subgroups. (M–O) indicates obesity group and its male and female subgroups. The relatively narrow 95% confidence intervals indicated that the accuracy of the symptom networks were good for all groups. Figure S2: Nonparametric bootstrapped confidence intervals of estimated edges in symptom networks. The red line represents the estimated edge, while the dark area indicates the 95% bootstrap confidence interval. (A–C) indicates all Chinese adults, adult males, and adult females in aged 19–65. (D–F) indicates underweight group and its male and female subgroups. (G–I) indicates normal weight group and its male and female subgroups. (J–L) indicates overweight group and its male and female subgroups. (M–O) indicates obesity group and its male and female subgroups. Edges in the networks were robust and can be trusted. Figure S3: Bootstrapped stability test for edge‐weight in symptom networks. The results of the bootstrapped difference tests (α = 0.05) for edge‐weights were shown in this figure. The color of the boxes indicates whether edge‐weights differ significantly from each other (i.e., black) or do not differ significantly (i.e., gray). The diagonal line indicates the strength of edge‐weights, shifting from red (negative associations) to white (representing weaker edges) and ultimately blue (representing stronger edge‐weights). (A–C) i [file PCHJ-15-e70104-s001.docx]

**Supplementary material A**

**Sensitivity Analysis Using Polychoric Correlations**

Because PHQ-9 and GAD-7 items are four-point Likert-type ordinal variables, polychoric correlations are theoretically preferable as they estimate associations between underlying continuous latent response variables. To evaluate the robustness of our primary Spearman-based network results, we conducted a sensitivity analysis using polychoric correlations under the same regularization framework and tuning parameter (γ = 0.5).

All analyses were conducted in R using the bootnet package (v1.5). The estimation procedure mirrored the primary analysis except for the correlation method.

**1. Data Preparation**

All questionnaire items were converted to numeric format and organized into a matrix:

| Data1 <- read_excel("F:/PBICR/PBICR-2021-obesity.xlsx", sheet = "all")  Data1[] <- lapply(Data1, function(x) as.numeric(as.character(x)))  Data1 <- as.matrix(Data1)  network1 <- estimateNetwork(Data1, default = "EBICglasso", tuning = 0.5, corMethod = "cor_auto" , corArgs = list(forcePD = FALSE, detectOrdinal=TRUE), threshold = TRUE) |
| --- |

**2. Polychoric Correlation Estimation**

Networks were estimated using the EBIC graphical LASSO model with automatic detection of ordinal variables:

| network1 <- estimateNetwork(Data1, default = "EBICglasso", tuning = 0.5, corMethod = "cor_auto" , corArgs = list(forcePD = TRUE, detectOrdinal=TRUE), threshold = TRUE) |
| --- |

- corMethod = "cor_auto" with detectOrdinal = TRUE ensures ordinal variables are automatically processed using polychoric correlations.
- During initial estimation, the polychoric correlation matrix was not positive definite. Therefore, forcePD = TRUE was applied to enforce positive definiteness.

**3. Technical Issues Encountered**

Although networks could be estimated after enforcing positive definiteness, errors occurred during Network Comparison Test (NCT) procedures:

| Error in lavCor (...): lavaan ERROR: polychoric correlation matrix could not be computed. |
| --- |

This issue likely reflects sparse endorsement in extreme response categories. During NCT permutation sampling, some resampled contingency tables became excessively sparse, making polychoric estimation unstable or infeasible. This phenomenon is consistent with methodological literature indicating that sparse ordinal distributions can destabilize polychoric correlation matrices and produce non-positive definite solutions.

Item-level frequency tables confirmed low endorsement rates in extreme categories across several BMI × gender strata, supporting this interpretation.

**4. Comparison of Network Structure**

Despite computational instability in resampling procedures, we successfully estimated polychoric-based networks and compared them to the original Spearman-based networks.

**4.1 Core Symptoms**

Although minor variations in centrality rankings were observed, there was substantial overlap in influential nodes across methods.

| Group | Original (Spearman) | Redo (Polychoric) |
| --- | --- | --- |
| Overall | GAD2, PHQ8, GAD4 | GAD2, GAD5, PHQ8 |
| Underweight | GAD5, GAD7, GAD2 | GAD5, GAD7, GAD2 |
| Normal | GAD7, PHQ8, GAD4 | GAD5, GAD2, PHQ8 |
| Overweight | GAD5, GAD2, PHQ4 | GAD2, PHQ8, GAD5 |
| Obesity | GAD4, GAD1, PHQ4 | GAD5, GAD4, PHQ8 |

Across groups, highly overlapping sets of anxiety-related nodes (particularly GAD2 and GAD5) consistently emerged as central.

**4.2 Strongest Edges**

The strongest edge was identical in several groups:

| Group | Original (Spearman) | Redo (Polychoric) |
| --- | --- | --- |
| Overall | GAD5-GAD7 | GAD5-GAD7 |
| Underweight | GAD5-GAD7 | GAD5-GAD7 |
| Normal | GAD5-GAD7 | GAD5-GAD7 |
| Overweight | GAD5-GAD7 | GAD1-GAD2 |
| Obesity | PHQ5-GAD3 | GAD3-GAD4 |

The edge GAD5–GAD7 remained the strongest association in most BMI categories under both methods.

**5. Interpretation**

Although polychoric correlations are theoretically optimal for ordinal data, sparse category endorsement in several strata resulted in computational instability, including non-positive definite matrices and failure of polychoric estimation during permutation-based NCT procedures.

Importantly:

- Core symptoms showed substantial overlap across estimation methods.
- The strongest edges were largely consistent.

Taken together, these findings indicate that the main conclusions are robust to the choice of correlation estimator. Given the sparse ordinal distributions and instability of polychoric estimation in resampling contexts, **the use of regularized Spearman partial correlations represents a statistically stable and methodologically defensible approach for the present data**.

**Supplementary material B**

**Sensitivity analysis: size-matched subsampling NCT**

Because BMI-by-gender subgroups were imbalanced in sample size, NCT results may be affected by reduced precision in the smaller subgroup. To assess the robustness of NCT findings against this imbalance, we conducted a sensitivity analysis using size-matched subsampling.

All analyses were conducted in R using the NetworkComparisonTest package. For each pairwise comparison, we treated the smaller subgroup as the reference size (n = nsmall). We repeatedly (1,000 iterations) randomly sampled nsmall participants without replacement from the larger subgroup, (re-)estimated networks using EBICglasso (Spearman correlations; pairwise complete observations; tuning parameter γ = 0.5; thresholding enabled), and performed NCT between the subsampled large-group network and the original small-group network. For each iteration we recorded the p-values for network structure invariance and global strength invariance, and (if applicable) centrality invariance based on expected influence with FDR correction. The detailed results see Table S10-S13.

| Data1 <- read_excel("F:/PBICR/PBICR-2021-underweight.xlsx", sheet = "male") # import Data  Data1[] <- lapply(Data1, function(x) as.numeric(as.character(x))) # Convert text to numeric  Data1 <- as.matrix(Data1) # Convert data to matrix  network1 <- estimateNetwork(Data1, default = "EBICglasso", tuning = 0.5, corMethod = "cor" , corArgs = list(method = "spearman", use = "pairwise.complete.obs"), threshold = TRUE)  Data3 <- read_excel("F:/PBICR/PBICR-2021-obesity.xlsx", sheet = "male")  Data3[] <- lapply(Data3, function(x) as.numeric(as.character(x)))  Data3 <- as.matrix(Data3)  network3 <- estimateNetwork(Data3, default = "EBICglasso", tuning = 0.5, corMethod = "cor" , corArgs = list(method = "spearman", use = "pairwise.complete.obs"), threshold = TRUE)  cov_cols <- 2:7  cov_names <- c("Gender","Age","Distribution","Education","Disease","BMI")  make_bins <- function(x, k = 4){  br <- unique(as.numeric(quantile(x, probs = seq(0,1,length.out=k+1), na.rm=TRUE)))  cut(x, breaks = br, include.lowest = TRUE, ordered_result = TRUE)  }  stratified_subsample_preserve_cat <- function(df, n_target, cov_cols, seed = NULL){  if(!is.null(seed)) set.seed(seed)  stopifnot(n_target <= nrow(df))    d <- as.data.frame(df)    # strata key：  cov_mat <- d[, cov_cols, drop=FALSE]  cov_mat <- lapply(cov_mat, function(x){  x <- as.character(x)  x[is.na(x)] <- "NA"  x  })  strata <- do.call(paste, c(cov_mat, sep = " \| "))  d$.strata <- strata    tab <- table(d$.strata)  props <- as.numeric(tab) / sum(tab)    n_floor <- floor(props * n_target)  remainder <- props * n_target - n_floor  need <- n_target - sum(n_floor)  if(need > 0){  add_idx <- order(remainder, decreasing = TRUE)[seq_len(need)]  n_floor[add_idx] <- n_floor[add_idx] + 1  }    n_alloc <- pmin(n_floor, as.numeric(tab))    short <- n_target - sum(n_alloc)  if(short > 0){  cap_left <- as.numeric(tab) - n_alloc  eligible <- which(cap_left > 0)  extra <- sample(eligible, size = short, replace = TRUE, prob = cap_left[eligible])  add_counts <- table(extra)  n_alloc[as.integer(names(add_counts))] <- n_alloc[as.integer(names(add_counts))] + as.integer(add_counts)  n_alloc <- pmin(n_alloc, as.numeric(tab))  }    strata_levels <- names(tab)  picked <- integer(0)  for(i in seq_along(strata_levels)){  ni <- n_alloc[i]  if(ni <= 0) next  rows <- which(d$.strata == strata_levels[i])  picked <- c(picked, sample(rows, ni, replace = FALSE))  }    if(length(picked) < n_target){  remain <- setdiff(seq_len(nrow(d)), picked)  picked <- c(picked, sample(remain, n_target - length(picked), replace = FALSE))  }    picked  }  ### Network Comparison Test by subsampling ###  n_small <- nrow(Data3) # The group with smaller people  idx_big_all <- 1:nrow(Data1)  idx <- sample(idx_big_all, n_small, replace=FALSE)  Data1_sub <- Data1[idx, ]  net1_sub <- estimateNetwork(Data1_sub, default = "EBICglasso", tuning = 0.5, corMethod = "cor" , corArgs = list(method = "spearman", use = "pairwise.complete.obs"), threshold = TRUE)  net2_sub <- estimateNetwork(Data3, default = "EBICglasso", tuning = 0.5, corMethod = "cor" , corArgs = list(method = "spearman", use = "pairwise.complete.obs"), threshold = TRUE)    nct_b <- NCT(net1_sub, net2_sub, gamma=0.5, it=1000,  test.edges=TRUE, test.centrality=TRUE,  centrality=c("expectedInfluence"),  p.adjust.methods="fdr")  p_struct <- nct_b$nwinv.pval  p_strength <- nct_b$glstrinv.pval  summary_list[] <- capture.output(summary(nct_b))  } |
| --- |

**Figure captions**

**Fig. S1. The accuracy of the symptom networks’ EI.** The x-axis indicates the percentage of cases of the original sample included at each step. The y-axis indicates the average of correlations between the centrality indices from the original network and the centrality indices from the networks that were re-estimated after excluding increasing percentages of cases. (A)~(C) indicates all Chinese adults, adult males, and adult females in aged 19~65. (D)~(F) indicates underweight group and its male and female subgroups. (G)~(I) indicates normal weight group and its male and female subgroups. (J)~(L) indicates overweight group and its male and female subgroups. (M)~(O) indicates obesity group and its male and female subgroups. The relatively narrow 95% confidence intervals indicated that the accuracy of the symptom networks were good for all groups.

**Fig. S2. Nonparametric bootstrapped confidence intervals of estimated edges in symptom networks.** The red line represents the estimated edge, while the dark area indicates the 95% bootstrap confidence interval. (A)~(C) indicates all Chinese adults, adult males, and adult females in aged 19~65. (D)~(F) indicates underweight group and its male and female subgroups. (G)~(I) indicates normal weight group and its male and female subgroups. (J)~(L) indicates overweight group and its male and female subgroups. (M)~(O) indicates obesity group and its male and female subgroups. Edges in the networks were robust and can be trusted.

**Fig. S3. Bootstrapped stability test for edge-weight in symptom networks.** The results of the bootstrapped difference tests (*α* = 0.05) for edge-weights were shown in this figure. The color of the boxes indicates whether edge-weights differ significantly from each other (i.e., black) or do not differ significantly (i.e., grey). The diagonal line indicates the strength of edge-weights, shifting from red (negative associations) to white (representing weaker edges) and ultimately blue (representing stronger edge-weights). (A)~(C) indicates all Chinese adults, adult males, and adult females in aged 19~65. (D)~(F) indicates underweight group and its male and female subgroups. (G)~(I) indicates normal weight group and its male and female subgroups. (J)~(L) indicates overweight group and its male and female subgroups. (M)~(O) indicates obesity group and its male and female subgroups.

**Fig. S4. Nonparametric bootstrapped difference test for nodes in symptom networks.** Grey boxes indicate no significant difference, whereas black boxes indicate a statistically significant difference (*p* < 0.05). Diagonal color and saturation represent the magnitude and direction of each estimated edge. (A)~(C) indicates all Chinese adults, adult males, and adult females in aged 19~65. (D)~(F) indicates underweight group and its male and female subgroups. (G)~(I) indicates normal weight group and its male and female subgroups. (J)~(L) indicates overweight group and its male and female subgroups. (M)~(O) indicates obesity group and its male and female subgroups.


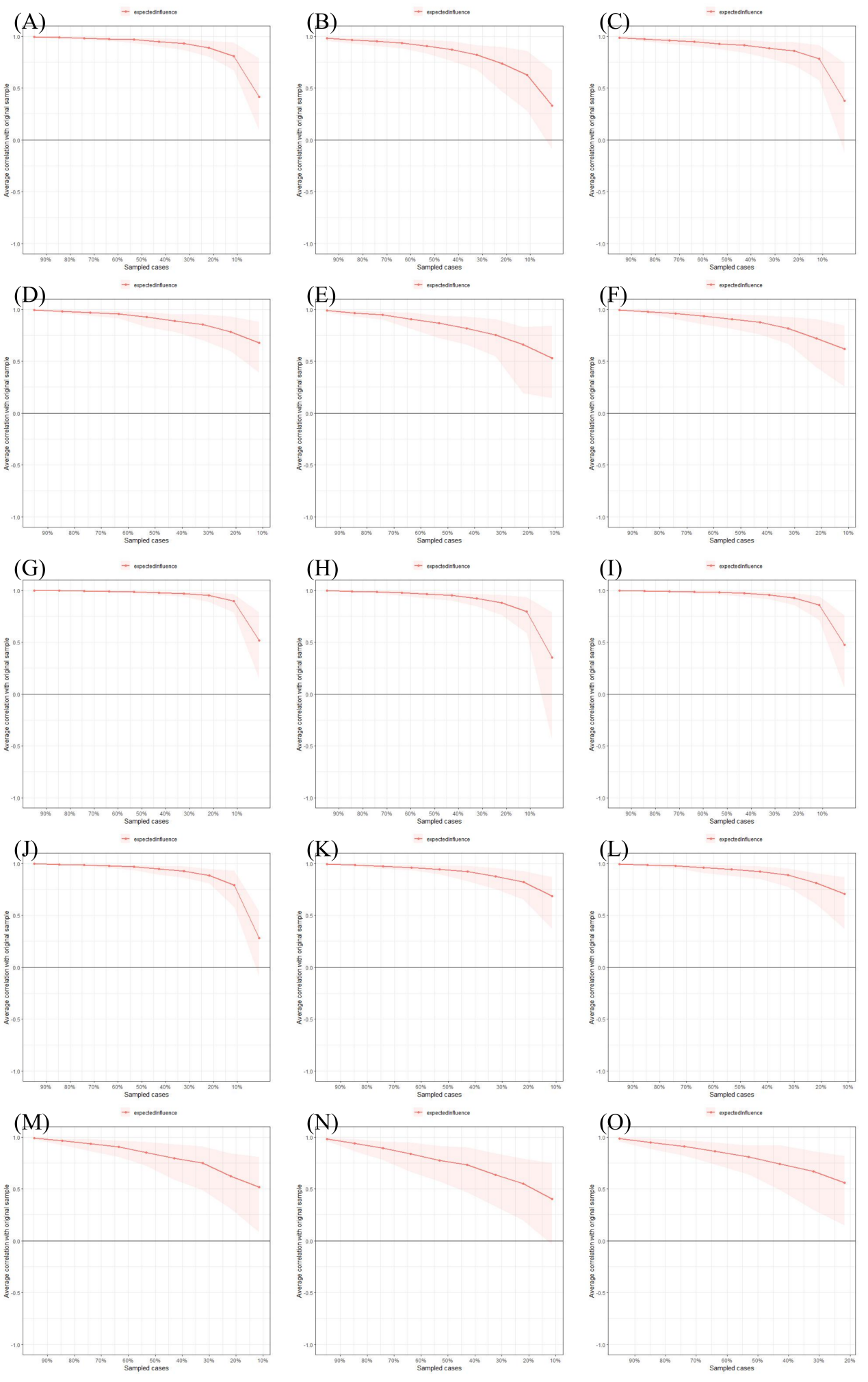


**Fig. S1. The accuracy of the symptom networks’ EI.** The x-axis indicates the percentage of cases of the original sample included at each step. The y-axis indicates the average of correlations between the centrality indices from the original network and the centrality indices from the networks that were re-estimated after excluding increasing percentages of cases. (A)~(C) indicates all Chinese adults, adult males, and adult females in aged 19~65. (D)~(F) indicates underweight group and its male and female subgroups. (G)~(I) indicates normal weight group and its male and female subgroups. (J)~(L) indicates overweight group and its male and female subgroups. (M)~(O) indicates obesity group and its male and female subgroups. The relatively narrow 95% confidence intervals indicated that the accuracy of the symptom networks were good for all groups.


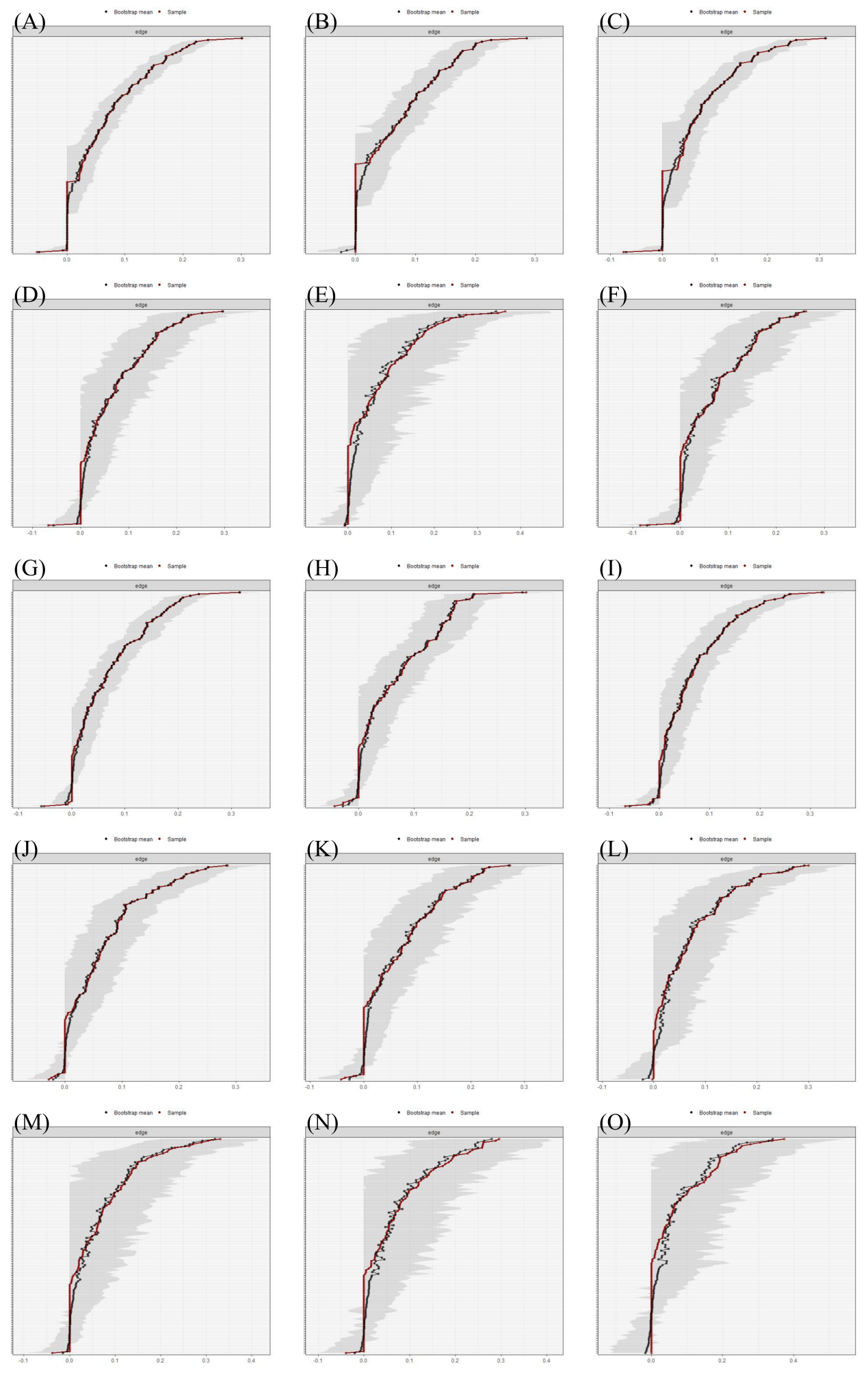


**Fig. S2. Nonparametric bootstrapped confidence intervals of estimated edges in symptom networks.** The red line represents the estimated edge, while the dark area indicates the 95% bootstrap confidence interval. (A)~(C) indicates all Chinese adults, adult males, and adult females in aged 19~65. (D)~(F) indicates underweight group and its male and female subgroups. (G)~(I) indicates normal weight group and its male and female subgroups. (J)~(L) indicates overweight group and its male and female subgroups. (M)~(O) indicates obesity group and its male and female subgroups. Edges in the networks were robust and can be trusted.


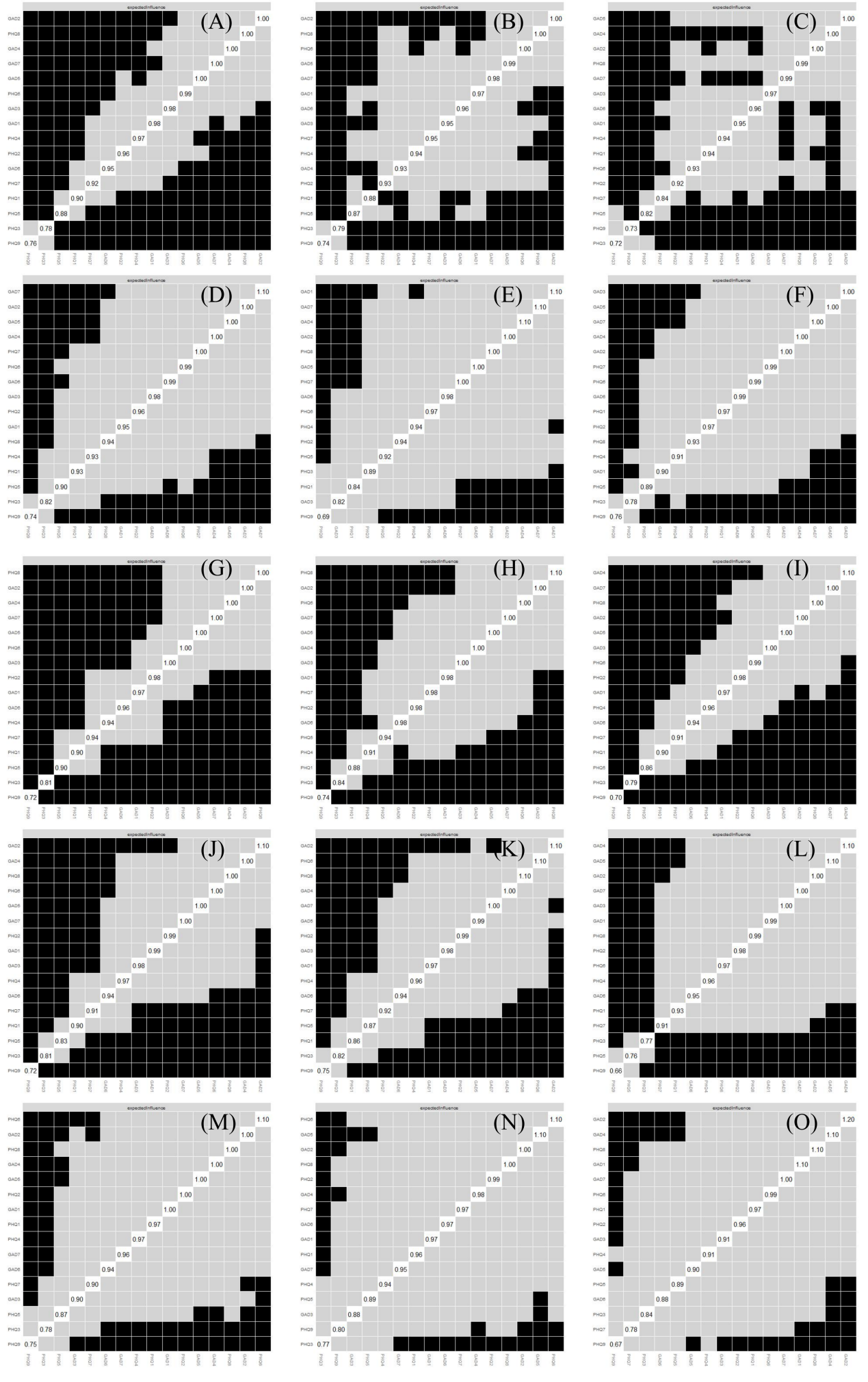


**Fig. S3. Bootstrapped stability test for edge-weight in symptom networks.** The results of the bootstrapped difference tests (*α* = 0.05) for edge-weights were shown in this figure. The color of the boxes indicates whether edge-weights differ significantly from each other (i.e., black) or do not differ significantly (i.e., grey). The diagonal line indicates the strength of edge-weights, shifting from red (negative associations) to white (representing weaker edges) and ultimately blue (representing stronger edge-weights). (A)~(C) indicates all Chinese adults, adult males, and adult females in aged 19~65. (D)~(F) indicates underweight group and its male and female subgroups. (G)~(I) indicates normal weight group and its male and female subgroups. (J)~(L) indicates overweight group and its male and female subgroups. (M)~(O) indicates obesity group and its male and female subgroups.


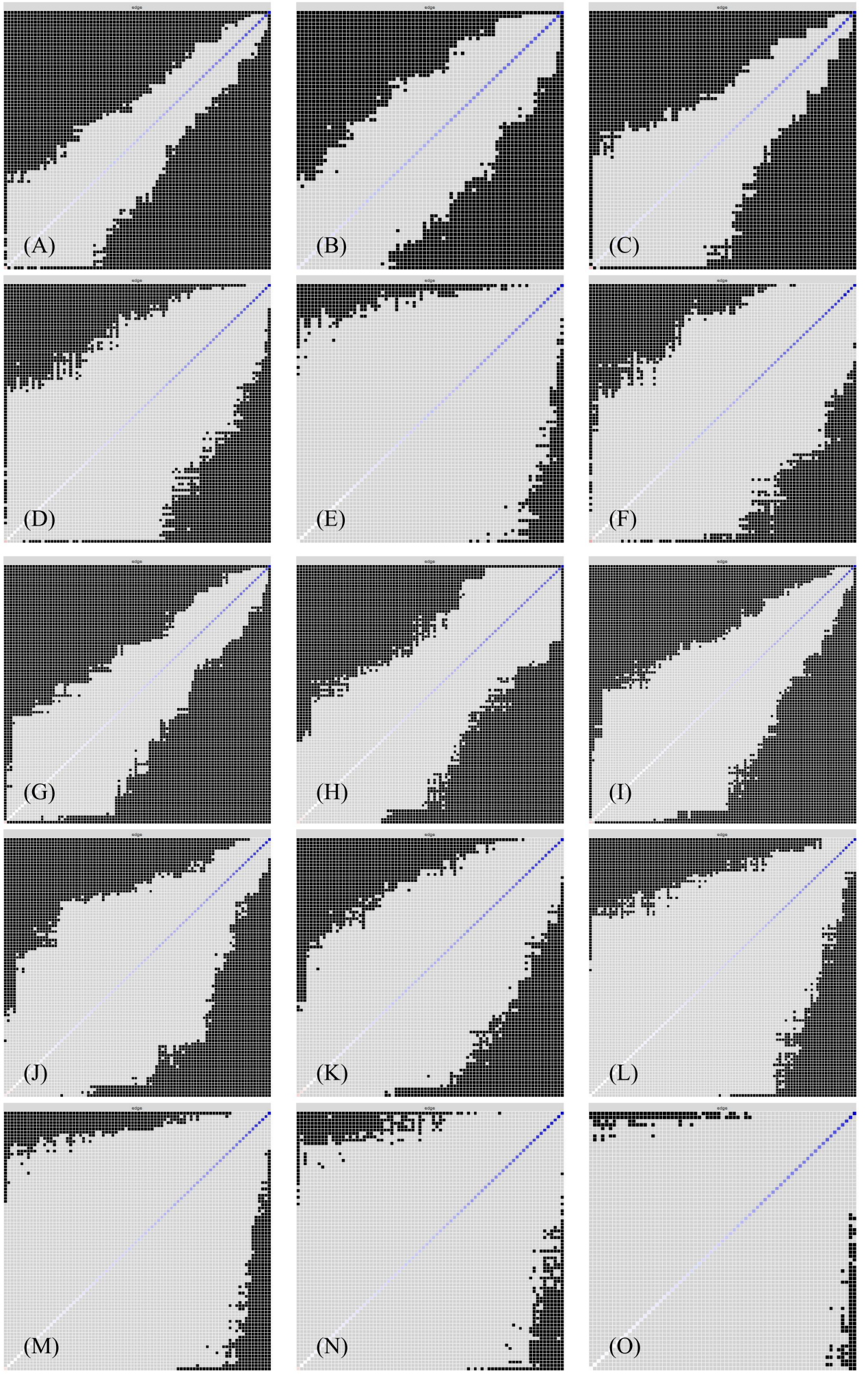


**Fig. S4. Nonparametric bootstrapped difference test for nodes in symptom networks.** Grey boxes indicate no significant difference, whereas black boxes indicate a statistically significant difference (*p* < 0.05). Diagonal color and saturation represent the magnitude and direction of each estimated edge. (A)~(C) indicates all Chinese adults, adult males, and adult females in aged 19~65. (D)~(F) indicates underweight group and its male and female subgroups. (G)~(I) indicates normal weight group and its male and female subgroups. (J)~(L) indicates overweight group and its male and female subgroups. (M)~(O) indicates obesity group and its male and female subgroups.

**Table captions**

**Table S1.** Distribution of BMI subgroups and associated levels of depression and anxiety (Overall)

**Table S2.** Distribution of BMI subgroups and associated levels of depression and anxiety (Male)

**Table S3.** Distribution of BMI subgroups and associated levels of depression and anxiety (Female)

**Table S4.** Weighted adjacency matrix of all participants

**Table S5.** Weighted adjacency matrix of underweight participants

**Table S6.** Weighted adjacency matrix of normal weight participants

**Table S7.** Weighted adjacency matrix of overweight participants

**Table S8.** Weighted adjacency matrix of obese participants

**Table S9.** Node centrality’s stability of network in the full sample and different BMI subgroups

**Table S10.** Network comparison results between different BMI subgroups using subsampling data

**Table S11.** Network comparison results between male subjects across Different BMI subgroups using subsampling data

**Table S12.** Network comparison results between female subjects across Different BMI subgroups using subsampling data

**Table S13.** Network comparison results between male and female subjects in the full sample or BMI subgroups using subsampling data

**Table S1.** Distribution of BMI subgroups and associated levels of depression and anxiety (Overall).

| Severity  BMI | Overall | Underweight | Normal weight | Overweight | Obesity | *P* value^[[1]](#footnote-1)^ |
| --- | --- | --- | --- | --- | --- | --- |
| Depression severity, n (%) |  |  |  |  |  | **< 0.001** |
| No depression | 4100 (45.10) | 434 (39.06) | 2596 (46.09) | 899 (45.52) | 171 (45.84) |  |
| Mild depression | 3159 (34.75) | 384 (34.56) | 1946 (34.55) | 705 (35.70) | 124 (33.24) |  |
| Moderate depression | 930 (10.23) | 159 (14.31) | 549 (9.75) | 188 (9.52) | 34 (9.12) |  |
| Severe depression | 902 (9.92) | 134 (12.06) | 541 (9.61) | 183 (9.27) | 44 (11.80) |  |
| Anxiety severity, n (%) |  |  |  |  |  | 0.090 |
| No anxiety | 5029 (55.32) | 573 (51.58) | 3137 (55.70) | 1106 (56.00) | 213 (57.10) |  |
| Mild anxiety | 2805 (30.85) | 361 (32.49) | 1706 (30.29) | 623 (31.54) | 115 (30.83) |  |
| Moderate anxiety | 1000 (11.00) | 132 (11.88) | 642 (11.40) | 194 (9.82) | 32 (8.58) |  |
| Severe anxiety | 257 (2.83) | 45 (4.05) | 147 (2.61) | 52 (2.63) | 13 (3.49) |  |

**Table S2.** Distribution of BMI subgroups and associated levels of depression and anxiety (Male).

| Severity  BMI | Overall | Underweight | Normal weight | Overweight | Obesity | *P* value^[[2]](#footnote-2)^ |
| --- | --- | --- | --- | --- | --- | --- |
| Depression severity, male |  |  |  |  |  | 0.058 |
| No depression | 1926 (45.04) | 134 (39.03) | 1145 (46.09) | 530 (45.52) | 117 (45.84) |  |
| Mild depression | 1335 (31.19) | 85 (25.15) | 788 (31.96) | 383 (32.61) | 79 (30.54) |  |
| Moderate depression | 416 (9.73) | 47 (14.31) | 232 (9.51) | 111 (9.43) | 26 (10.14) |  |
| Severe depression | 473 (11.04) | 44 (13.20) | 280 (11.34) | 117 (9.82) | 32 (12.44) |  |
| Anxiety severity, male |  |  |  |  |  | **0.044** |
| No anxiety | 2309 (53.98) | 169 (51.58) | 1355 (55.70) | 643 (56.00) | 142 (57.10) |  |
| Mild anxiety | 1197 (27.99) | 80 (24.24) | 694 (28.33) | 344 (30.01) | 79 (31.88) |  |
| Moderate anxiety | 513 (11.99) | 43 (12.95) | 326 (13.38) | 118 (10.26) | 26 (10.37) |  |
| Severe anxiety | 131 (3.06) | 18 (5.45) | 70 (2.85) | 36 (3.12) | 7 (2.83) |  |

**Table S3.** Distribution of BMI subgroups and associated levels of depression and anxiety (Female).

| Severity  BMI | Overall | Underweight | Normal weight | Overweight | Obesity | *P* value^[[3]](#footnote-3)^ |
| --- | --- | --- | --- | --- | --- | --- |
| Depression severity, female |  |  |  |  |  | **<0.001** |
| No depression | 2174 (50.94) | 300 (50.00) | 1451 (51.60) | 369 (52.53) | 54 (37.50) |  |
| Mild depression | 1824 (42.66) | 299 (49.83) | 1158 (42.82) | 322 (45.73) | 45 (31.25) |  |
| Moderate depression | 514 (12.03) | 112 (18.64) | 317 (11.54) | 77 (10.89) | 8 (5.56) |  |
| Severe depression | 429 (10.04) | 90 (15.00) | 261 (9.56) | 66 (9.42) | 12 (8.33) |  |
| Anxiety severity, female |  |  |  |  |  | **0.039** |
| No anxiety | 2720 (63.70) | 404 (66.67) | 1782 (63.76) | 463 (66.60) | 71 (49.30) |  |
| Mild anxiety | 1608 (37.57) | 281 (46.83) | 1012 (36.82) | 279 (40.09) | 36 (25.35) |  |
| Moderate anxiety | 487 (11.41) | 89 (14.83) | 316 (11.55) | 76 (11.04) | 6 (4.17) |  |
| Severe anxiety | 126 (2.95) | 27 (4.50) | 77 (2.82) | 16 (2.31) | 6 (4.17) |  |

**Table S4.** Weighted adjacency matrix of all participants

|  | PAQ1 | PAQ2 | PAQ3 | PAQ4 | PAQ5 | PAQ6 | PAQ7 | PAQ8 | PAQ9 | GAD1 | GAD2 | GAD3 | GAD4 | GAD5 | GAD6 | GAD7 |
| --- | --- | --- | --- | --- | --- | --- | --- | --- | --- | --- | --- | --- | --- | --- | --- | --- |
| PHQ1 | 0.000 |  |  |  |  |  |  |  |  |  |  |  |  |  |  |  |
| PHQ2 | 0.199 | 0.000 |  |  |  |  |  |  |  |  |  |  |  |  |  |  |
| PHQ3 | 0.108 | 0.080 | 0.000 |  |  |  |  |  |  |  |  |  |  |  |  |  |
| PHQ4 | 0.222 | 0.138 | 0.243 | 0.000 |  |  |  |  |  |  |  |  |  |  |  |  |
| PHQ5 | 0.082 | 0.027 | 0.141 | 0.113 | 0.000 |  |  |  |  |  |  |  |  |  |  |  |
| PHQ6 | 0.068 | 0.149 | 0.000 | 0.069 | 0.092 | 0.000 |  |  |  |  |  |  |  |  |  |  |
| PHQ7 | 0.111 | 0.025 | 0.050 | 0.065 | 0.147 | 0.165 | 0.000 |  |  |  |  |  |  |  |  |  |
| PHQ8 | 0.000 | 0.089 | 0.056 | 0.000 | 0.064 | 0.171 | 0.196 | 0.000 |  |  |  |  |  |  |  |  |
| PHQ9 | 0.000 | 0.068 | 0.000 | 0.000 | 0.079 | 0.096 | 0.053 | 0.219 | 0.000 |  |  |  |  |  |  |  |
| GAD1 | 0.107 | 0.054 | 0.000 | 0.024 | 0.067 | 0.031 | 0.000 | 0.000 | 0.000 | 0.000 |  |  |  |  |  |  |
| GAD2 | 0.000 | 0.044 | 0.000 | 0.000 | 0.000 | 0.081 | 0.000 | 0.024 | 0.026 | 0.188 | 0.000 |  |  |  |  |  |
| GAD3 | 0.000 | 0.000 | 0.051 | 0.026 | 0.000 | 0.046 | 0.022 | 0.000 | 0.000 | 0.168 | 0.170 | 0.000 |  |  |  |  |
| GAD4 | 0.000 | 0.000 | 0.021 | 0.072 | 0.000 | 0.022 | 0.021 | 0.000 | 0.000 | 0.122 | 0.207 | 0.210 | 0.000 |  |  |  |
| GAD5 | 0.000 | 0.000 | 0.000 | -0.052 | 0.043 | 0.000 | 0.037 | 0.137 | 0.036 | 0.087 | 0.075 | 0.060 | 0.137 | 0.000 |  |  |
| GAD6 | 0.000 | 0.048 | 0.032 | 0.052 | 0.030 | 0.000 | 0.032 | 0.000 | 0.000 | 0.107 | 0.082 | 0.127 | 0.171 | 0.148 | 0.000 |  |
| GAD7 | 0.000 | 0.040 | 0.000 | 0.000 | 0.000 | 0.000 | 0.000 | 0.068 | 0.181 | 0.020 | 0.141 | 0.104 | 0.035 | 0.302 | 0.123 | 0.000 |

**Table S5.** Weighted adjacency matrix of underweight participants

|  | PAQ1 | PAQ2 | PAQ3 | PAQ4 | PAQ5 | PAQ6 | PAQ7 | PAQ8 | PAQ9 | GAD1 | GAD2 | GAD3 | GAD4 | GAD5 | GAD6 | GAD7 |
| --- | --- | --- | --- | --- | --- | --- | --- | --- | --- | --- | --- | --- | --- | --- | --- | --- |
| PHQ1 | 0.000 |  |  |  |  |  |  |  |  |  |  |  |  |  |  |  |
| PHQ2 | 0.196 | 0.000 |  |  |  |  |  |  |  |  |  |  |  |  |  |  |
| PHQ3 | 0.116 | 0.071 | 0.000 |  |  |  |  |  |  |  |  |  |  |  |  |  |
| PHQ4 | 0.211 | 0.180 | 0.219 | 0.000 |  |  |  |  |  |  |  |  |  |  |  |  |
| PHQ5 | 0.000 | 0.000 | 0.230 | 0.115 | 0.000 |  |  |  |  |  |  |  |  |  |  |  |
| PHQ6 | 0.074 | 0.126 | 0.072 | 0.138 | 0.066 | 0.000 |  |  |  |  |  |  |  |  |  |  |
| PHQ7 | 0.165 | 0.072 | 0.000 | 0.000 | 0.198 | 0.142 | 0.000 |  |  |  |  |  |  |  |  |  |
| PHQ8 | 0.000 | 0.115 | 0.000 | 0.000 | 0.000 | 0.147 | 0.161 | 0.000 |  |  |  |  |  |  |  |  |
| PHQ9 | 0.000 | 0.000 | 0.000 | 0.000 | 0.000 | 0.094 | 0.098 | 0.172 | 0.000 |  |  |  |  |  |  |  |
| GAD1 | 0.063 | 0.000 | 0.000 | 0.054 | 0.055 | 0.000 | 0.000 | 0.000 | 0.000 | 0.000 |  |  |  |  |  |  |
| GAD2 | 0.000 | 0.058 | 0.000 | 0.000 | 0.000 | 0.090 | 0.000 | 0.000 | 0.000 | 0.136 | 0.000 |  |  |  |  |  |
| GAD3 | 0.000 | 0.000 | 0.000 | 0.000 | 0.000 | 0.000 | 0.125 | 0.000 | 0.000 | 0.214 | 0.151 | 0.000 |  |  |  |  |
| GAD4 | 0.000 | 0.047 | 0.000 | 0.051 | 0.000 | 0.000 | 0.000 | 0.000 | 0.000 | 0.078 | 0.257 | 0.155 | 0.000 |  |  |  |
| GAD5 | 0.000 | 0.000 | 0.052 | -0.098 | 0.000 | 0.000 | 0.000 | 0.168 | 0.087 | 0.164 | 0.079 | 0.105 | 0.063 | 0.000 |  |  |
| GAD6 | 0.063 | 0.000 | 0.000 | 0.000 | 0.000 | 0.000 | 0.000 | 0.000 | 0.000 | 0.118 | 0.087 | 0.128 | 0.227 | 0.122 | 0.000 |  |
| GAD7 | 0.000 | 0.000 | 0.000 | 0.000 | 0.000 | 0.000 | 0.000 | 0.085 | 0.185 | 0.000 | 0.154 | 0.065 | 0.084 | 0.301 | 0.146 | 0.000 |

**Table S6.** Weighted adjacency matrix of normal weight participants

|  | PAQ1 | PAQ2 | PAQ3 | PAQ4 | PAQ5 | PAQ6 | PAQ7 | PAQ8 | PAQ9 | GAD1 | GAD2 | GAD3 | GAD4 | GAD5 | GAD6 | GAD7 |
| --- | --- | --- | --- | --- | --- | --- | --- | --- | --- | --- | --- | --- | --- | --- | --- | --- |
| PHQ1 | 0.000 |  |  |  |  |  |  |  |  |  |  |  |  |  |  |  |
| PHQ2 | 0.191 | 0.000 |  |  |  |  |  |  |  |  |  |  |  |  |  |  |
| PHQ3 | 0.117 | 0.086 | 0.000 |  |  |  |  |  |  |  |  |  |  |  |  |  |
| PHQ4 | 0.205 | 0.143 | 0.240 | 0.000 |  |  |  |  |  |  |  |  |  |  |  |  |
| PHQ5 | 0.100 | 0.000 | 0.136 | 0.096 | 0.000 |  |  |  |  |  |  |  |  |  |  |  |
| PHQ6 | 0.076 | 0.153 | 0.000 | 0.062 | 0.090 | 0.000 |  |  |  |  |  |  |  |  |  |  |
| PHQ7 | 0.100 | 0.036 | 0.063 | 0.064 | 0.135 | 0.168 | 0.000 |  |  |  |  |  |  |  |  |  |
| PHQ8 | 0.000 | 0.079 | 0.049 | 0.000 | 0.064 | 0.173 | 0.194 | 0.000 |  |  |  |  |  |  |  |  |
| PHQ9 | 0.000 | 0.068 | 0.000 | 0.000 | 0.088 | 0.098 | 0.045 | 0.223 | 0.000 |  |  |  |  |  |  |  |
| GAD1 | 0.112 | 0.053 | 0.000 | 0.042 | 0.080 | 0.024 | 0.000 | 0.000 | 0.000 | 0.000 |  |  |  |  |  |  |
| GAD2 | 0.000 | 0.000 | 0.000 | 0.000 | 0.000 | 0.092 | 0.000 | 0.032 | 0.000 | 0.186 | 0.000 |  |  |  |  |  |
| GAD3 | 0.000 | 0.022 | 0.056 | 0.027 | 0.000 | 0.041 | 0.000 | 0.000 | 0.000 | 0.170 | 0.177 | 0.000 |  |  |  |  |
| GAD4 | 0.000 | 0.000 | 0.000 | 0.078 | 0.000 | 0.021 | 0.030 | 0.000 | 0.000 | 0.139 | 0.203 | 0.208 | 0.000 |  |  |  |
| GAD5 | 0.000 | 0.000 | 0.032 | -0.064 | 0.042 | 0.000 | 0.038 | 0.133 | 0.029 | 0.068 | 0.070 | 0.025 | 0.161 | 0.000 |  |  |
| GAD6 | 0.000 | 0.057 | 0.000 | 0.060 | 0.044 | 0.000 | 0.031 | 0.000 | 0.000 | 0.104 | 0.087 | 0.137 | 0.151 | 0.142 | 0.000 |  |
| GAD7 | 0.000 | 0.041 | 0.000 | 0.000 | 0.000 | 0.000 | 0.000 | 0.068 | 0.181 | 0.000 | 0.141 | 0.132 | 0.022 | 0.316 | 0.128 | 0.000 |

**Table S7.** Weighted adjacency matrix of overweight participants

|  | PAQ1 | PAQ2 | PAQ3 | PAQ4 | PAQ5 | PAQ6 | PAQ7 | PAQ8 | PAQ9 | GAD1 | GAD2 | GAD3 | GAD4 | GAD5 | GAD6 | GAD7 |
| --- | --- | --- | --- | --- | --- | --- | --- | --- | --- | --- | --- | --- | --- | --- | --- | --- |
| PHQ1 | 0.000 |  |  |  |  |  |  |  |  |  |  |  |  |  |  |  |
| PHQ2 | 0.209 | 0.000 |  |  |  |  |  |  |  |  |  |  |  |  |  |  |
| PHQ3 | 0.091 | 0.062 | 0.000 |  |  |  |  |  |  |  |  |  |  |  |  |  |
| PHQ4 | 0.253 | 0.102 | 0.247 | 0.000 |  |  |  |  |  |  |  |  |  |  |  |  |
| PHQ5 | 0.051 | 0.000 | 0.116 | 0.146 | 0.000 |  |  |  |  |  |  |  |  |  |  |  |
| PHQ6 | 0.000 | 0.164 | 0.066 | 0.070 | 0.087 | 0.000 |  |  |  |  |  |  |  |  |  |  |
| PHQ7 | 0.108 | 0.000 | 0.057 | 0.073 | 0.140 | 0.164 | 0.000 |  |  |  |  |  |  |  |  |  |
| PHQ8 | 0.000 | 0.090 | 0.000 | 0.000 | 0.091 | 0.155 | 0.212 | 0.000 |  |  |  |  |  |  |  |  |
| PHQ9 | 0.000 | 0.103 | 0.000 | 0.000 | 0.063 | 0.094 | 0.054 | 0.234 | 0.000 |  |  |  |  |  |  |  |
| GAD1 | 0.095 | 0.059 | 0.000 | 0.000 | 0.000 | 0.076 | 0.000 | 0.000 | 0.000 | 0.000 |  |  |  |  |  |  |
| GAD2 | 0.000 | 0.099 | 0.000 | 0.000 | 0.000 | 0.036 | 0.000 | 0.043 | 0.000 | 0.210 | 0.000 |  |  |  |  |  |
| GAD3 | 0.000 | 0.000 | 0.063 | 0.039 | 0.000 | 0.065 | 0.000 | 0.000 | 0.000 | 0.142 | 0.187 | 0.000 |  |  |  |  |
| GAD4 | 0.000 | 0.000 | 0.055 | 0.052 | 0.000 | 0.000 | 0.000 | 0.000 | 0.000 | 0.106 | 0.192 | 0.225 | 0.000 |  |  |  |
| GAD5 | 0.000 | 0.000 | 0.000 | 0.000 | 0.048 | 0.000 | 0.055 | 0.124 | 0.000 | 0.106 | 0.081 | 0.094 | 0.107 | 0.000 |  |  |
| GAD6 | 0.062 | 0.000 | 0.046 | 0.049 | 0.000 | 0.000 | 0.000 | 0.000 | 0.000 | 0.106 | 0.071 | 0.102 | 0.192 | 0.156 | 0.000 |  |
| GAD7 | 0.000 | 0.000 | 0.000 | 0.000 | 0.000 | 0.000 | 0.000 | 0.051 | 0.187 | 0.076 | 0.127 | 0.070 | 0.038 | 0.287 | 0.093 | 0.000 |

**Table S8.** Weighted adjacency matrix of obese participants

|  | PAQ1 | PAQ2 | PAQ3 | PAQ4 | PAQ5 | PAQ6 | PAQ7 | PAQ8 | PAQ9 | GAD1 | GAD2 | GAD3 | GAD4 | GAD5 | GAD6 | GAD7 |
| --- | --- | --- | --- | --- | --- | --- | --- | --- | --- | --- | --- | --- | --- | --- | --- | --- |
| PHQ1 | 0.000 |  |  |  |  |  |  |  |  |  |  |  |  |  |  |  |
| PHQ2 | 0.224 | 0.000 |  |  |  |  |  |  |  |  |  |  |  |  |  |  |
| PHQ3 | 0.000 | 0.108 | 0.000 |  |  |  |  |  |  |  |  |  |  |  |  |  |
| PHQ4 | 0.342 | 0.106 | 0.298 | 0.000 |  |  |  |  |  |  |  |  |  |  |  |  |
| PHQ5 | 0.102 | 0.000 | 0.000 | 0.135 | 0.000 |  |  |  |  |  |  |  |  |  |  |  |
| PHQ6 | 0.104 | 0.000 | 0.000 | 0.000 | 0.193 | 0.000 |  |  |  |  |  |  |  |  |  |  |
| PHQ7 | 0.000 | 0.000 | 0.000 | 0.116 | 0.163 | 0.193 | 0.000 |  |  |  |  |  |  |  |  |  |
| PHQ8 | 0.000 | 0.000 | 0.194 | 0.000 | 0.000 | 0.288 | 0.142 | 0.000 |  |  |  |  |  |  |  |  |
| PHQ9 | 0.000 | 0.148 | 0.000 | 0.000 | 0.000 | 0.000 | 0.000 | 0.239 | 0.000 |  |  |  |  |  |  |  |
| GAD1 | 0.091 | 0.000 | 0.000 | 0.000 | 0.130 | 0.000 | 0.000 | 0.000 | 0.000 | 0.000 |  |  |  |  |  |  |
| GAD2 | 0.000 | 0.000 | 0.000 | 0.000 | 0.000 | 0.000 | 0.000 | 0.099 | 0.125 | 0.221 | 0.000 |  |  |  |  |  |
| GAD3 | 0.000 | 0.000 | 0.000 | 0.000 | -0.104 | 0.083 | 0.000 | 0.000 | 0.000 | 0.126 | 0.000 | 0.000 |  |  |  |  |
| GAD4 | 0.000 | 0.000 | 0.000 | 0.000 | 0.095 | 0.082 | 0.000 | 0.000 | 0.000 | 0.143 | 0.151 | 0.317 | 0.000 |  |  |  |
| GAD5 | 0.000 | 0.000 | 0.000 | 0.000 | 0.000 | 0.000 | 0.000 | 0.000 | 0.000 | 0.000 | 0.130 | 0.209 | 0.095 | 0.000 |  |  |
| GAD6 | 0.000 | 0.000 | 0.000 | 0.000 | 0.000 | 0.000 | 0.089 | 0.000 | 0.000 | 0.142 | 0.000 | 0.000 | 0.153 | 0.274 | 0.000 |  |
| GAD7 | 0.000 | 0.000 | 0.000 | 0.000 | 0.000 | 0.000 | 0.000 | 0.000 | 0.129 | 0.148 | 0.113 | 0.000 | 0.000 | 0.170 | 0.139 | 0.000 |

**Table S9.** Node centrality’s stability of network in the full sample and different BMI subgroups

| Total | CS | Male | CS | Female | CS |
| --- | --- | --- | --- | --- | --- |
| All | 0.781 | All | 0.572 | All | 0.781 |
| Underweight | 0.677 | Underweight | 0.468 | Underweight | 0.572 |
| Normal | 0.885 | Normal | 0.781 | Normal | 0.885 |
| Overweight | 0.781 | Overweight | 0.677 | Overweight | 0.676 |
| Obesity | 0.466 | Obesity | 0.362 | Obesity | 0.361 |

**Table S10.** Network comparison results between different BMI subgroups using subsampling data.

|  | **Network Structure invariance** | **Global Strength invariance** |
| --- | --- | --- |
| Underweight – Normal | *M* = 0.143  *p* = 0.297 | underweight = 4.515, normal = 4.533  *S* = 0.019  *p* = 0.837 |
| Underweight – Overweight | *M* = 0.146  *p* = 0.313 | underweight = 4.515, overweight = 4.463  *S* = 0.051  *p* = 0.621 |
| Underweight – Obesity | *M* = 0.251  *p* = 0.249 | underweight = 4.240, obesity = 4.005  *S* = 0.652  *p* = 0.502 |
| Normal – Overweight | *M* = 0.085  *p* = 0.721 | normal = 4.525, overweight = 4.524  *S* = 0.001  *p* = 0.995 |
| Normal – Obesity | *M* = 0.118  *p* = 0.816 | normal = 4.169, obesity = 4.005  *S* = 0.165  *p* = 0.474 |
| Overweight – Obesity | *M* = 0.285  *p* = 0.131 | overweight = 4.416, obesity = 4.005  *S* = 0.141  *p* = 0.508 |

*M*: the value of the maximum difference in edge weights; *S*: the value of difference in sum of all edge weights.

**Table S11.** Network comparison results between male subjects across Different BMI subgroups using subsampling data.

|  | **Network Structure invariance** | **Global Strength invariance** |
| --- | --- | --- |
| Underweight (male) – Normal (male) | *M* = 0.238  *p* = 0.573 | underweight = 4.052, normal = 4.210  *S* = 0.157  *p* = 0.440 |
| Underweight (male) – Overweight (male) | *M* = 0.243  *p* = 0.489 | underweight = 4.052, overweight = 4.159  *S* = 0.106  *p* = 0.679 |
| Underweight (male) – Obesity (male) | *M* = 0.377  ***p* = 0.039** | underweight = 4.440, obesity = 4.067  *S* = 0.373  *p* = 0.180 |
| Normal (male) – Overweight (male) | *M* = 0.151  *p* = 0.292 | normal = 4.528, overweight = 4.468  *S* = 0.061  *p* = 0.583 |
| Normal (male) – Obesity (male) | *M* = 0.484  ***p* = 0.003** | normal = 4.333, obesity = 4.067  *S* = 0.266  *p* = 0.400 |
| Overweight (male) – Obesity (male) | *M* = 0.317  *p* = 0.202 | overweight = 4.406, obesity = 4.067  *S* = 0.339  *p* = 0.313 |

**Table S12.** Network comparison results between female subjects across Different BMI subgroups using subsampling data.

|  | **Network Structure invariance** | **Global Strength invariance** |
| --- | --- | --- |
| Underweight (female) – Normal (female) | *M* = 0.147  *p* = 0.553 | underweight = 4.604, normal = 4.402  *S* = 0.202  *p* = 0.128 |
| Underweight (female) – Overweight (female) | *M* = 0.146  *p* = 0.656 | underweight = 4.604, overweight = 4.307  *S* = 0.297  ***p* = 0.026** |
| Underweight (female) – Obesity (female) | *M* = 0.447  *p* = 0.127 | underweight = 4.065, obesity = 4.831  *S* = 0.766  *p* = 0.289 |
| Normal (female) – Overweight (female) | *M* = 0.158  *p* = 0.284 | normal = 4.455, overweight = 4.359  *S* = 0.096  *p* = 0.486 |
| Normal (female) – Obesity (female) | *M* = 0.354  *p* = 0.644 | normal = 3.823, obesity = 4.831  *S* = 1.007  *p* = 0.120 |
| Overweight (female) – Obesity (female) | *M* = 0.305  *p* = 0.776 | overweight = 4.310, obesity = 4.831  *S* = 0.521  *p* = 0.348 |

**Table S13.** Network comparison results between male and female subjects in the full sample or BMI subgroups using subsampling data.

|  | **Network Structure invariance** | **Global Strength invariance** |
| --- | --- | --- |
| All (male) – All (female) | *M* = 0.061  *p* = 0.725 | male = 4.624, female = 4.540  *S* = 0.083  ***p* = 0.018** |
| Underweight (male) – Underweight (female) | *M* = 0.203  *p* = 0.792 | male = 4.052, female = 4.320  *S* = 0.268  *p* = 0.284 |
| Normal (male) – Normal (female) | *M* = 0.111  *p* = 0.116 | male = 4.601, female = 4.511  *S* = 0.090  *p* = 0.085 |
| Overweight (male) – Overweight (female) | *M* = 0.225  ***p* = 0.026** | male = 4.582, female = 4.359  *S* = 0.223  *p* = 0.054 |
| Obesity (male) – Obesity (female) | *M* = 0.447  *p* = 0.190 | male = 4.325, female = 4.831  *S* = 0.506  *p* = 0.438 |

1. *P* values were determined by the Chi-square test. [↑](#footnote-ref-1)
2. *P* values were determined by the Chi-square test. [↑](#footnote-ref-2)
3. *P* values were determined by the Chi-square test. [↑](#footnote-ref-3)
